# Safety classification of herbal medicines used in pregnancy in a multinational study

**DOI:** 10.1186/s12906-016-1079-z

**Published:** 2016-03-15

**Authors:** D. A. Kennedy, A. Lupattelli, G. Koren, H. Nordeng

**Affiliations:** The Motherisk Program, Division of Clinical Pharmacology and Toxicology, The Hospital for Sick Children, University of Toronto, 555 University Avenue, Toronto, ON M5G 1X8 Canada; PharmacoEpidemiology and Drug Safety Research Group, School of Pharmacy, PharmaTox Strategic Initiative, Faculty of Mathematics and Natural Sciences, University of Oslo, Postbox 1068 Blindern, 0316 Oslo, Norway; Division of Mental Health, Norwegian Institute of Public Health, Oslo, Norway

**Keywords:** Herbal medicine, Complementary and alternative medicine, Pregnancy, Safety, CAM

## Abstract

**Background:**

The use of herbal medicines for health prevention and ailments is an increasing trend worldwide. Women in pregnancy are no exception; the reported prevalence of herbal medicine use in pregnancy ranges from 1 to 60 %. Despite a common perception of safety, herbal medicines may have potent pharmacological actions, and historically, have been used for this reason.

**Methods:**

A multinational, cross-sectional study on how women treat disease and pregnancy-related health ailments was conducted between October 2011 and February 2012 in Europe, North America, and Australia. This study’s primary aim was to evaluate and classify the herbal medicines used according to their safety in pregnancy and, secondly, to investigate risk factors associated with the use of contraindicated herbal medicines during pregnancy.

**Results:**

In total, 29.3 % of the women (*n* = 2673) reported the use of herbal medicines in pregnancy; of which we were able to identify 126 specific herbal medicines used by 2379 women (89.0 %). Twenty seven out of 126 herbal medicines were classified as contraindicated in pregnancy, and were used by 476 women (20.0 %). Twenty-eight were classified as safe for use in pregnancy and used by the largest number of women (*n* = 1128, 47.4 %). The greatest number was classified as requiring caution in pregnancy; these sixty herbal medicines were used by 751 women (31.6 %). Maternal factors associated with the use of contraindicated herbal medicines in pregnancy were found to be working in the home, having a university education, not using folic acid, and consuming alcohol. Interestingly, the recommendation to take a contraindicated herbal medicine was three times more likely to be from a healthcare practitioner (HCP) than an informal source.

**Conclusion:**

Based on the current literature the majority of women in this study used an herbal medicine that was classified as safe for use in pregnancy. Women who reported taking a contraindicated herb were more likely to have been recommended it use by an HCP rather than informal source(s), indicating an urgent need for more education among HCPs. The paucity of human studies on herbal medicines safety in pregnancy stands in stark contrast to the widespread use of these products among pregnant women.

**Electronic supplementary material:**

The online version of this article (doi:10.1186/s12906-016-1079-z) contains supplementary material, which is available to authorized users.

## Background

The use of herbal medicines for health prevention and ailments is an increasing trend worldwide. A recent report on international herbal sales suggests that by 2015 sales are expected to reach $93 billion dollars, up from $33 billion in 2010 [1]. Women in pregnancy are no exception; a review of studies from the Western world, reported that the prevalence of herbal medicine use in pregnancy ranged from 1 to 60 % [2]. The prevalence rates were 34 % in Australia [3], 58 % in the United Kingdom [4], 40 % in Norway [5], 48 % in Italy [6] and 6–9 % in the US and Canada [7, 8]. In pregnancy, women often use herbal medicines due to the perception that these substances are more natural and therefore safer to use as compared to pharmaceutical medicines [2, 9–11]. Research suggests that in many instances, the use of herbal medicines speaks to a woman’s wish to have greater choice in their health and aligns with their desire for a holistic approach to their well-being [2, 12].

In most countries, herbal medicines are available as non-prescription medicines. Given such ease of access, most women report that the decision to use an herbal medicine came from either their own initiative or on the recommendation from family and/or friends. A small percentage of women do report that the recommendation to use an herbal medicine came from a healthcare practitioner (HCP) [2, 13, 14]. One study evaluated 400 different women’s knowledge regarding the indications for use of 10 specific herbal medicines (bearberry, dandelion, echinacea, ginkgo, hops, horsetail, lemon balm, St. John’s wort, sage, and valerian). These researchers found that over 78.3 % of the women surveyed had little knowledge regarding the indications for use of these herbal medicines, even though 31.3 % had used the herbal medicine in their pregnancy [10].

Despite this common perception of safety, herbal medicines may have potent pharmacological actions, and have, in fact, been used for centuries, for example as emmenagogues to promote abortion. Moreover, very little is known about the extent to which potentially harmful herbal medicines are used in pregnancy. Concerns range, with some herbal medicines, from teratogenicity to an increased risk of maternal bleeding or impact on neonatal hormones due to the hormonal nature of the herbal medicine [15]. In a previous study, 39 % of the women reporting having used herbal medicines during pregnancy had used herbal medicines that were considered possibly harmful or herbs where information about safety in pregnancy was missing [16].

This article is a continuation of the investigations of our previously published study on the use of herbal medicines in pregnancy among more than 9000 women across 18 countries and five regions of the world. More than one out of four women (28.9 %) reported using 127 different herbal medicines during pregnancy [13]. The objective of the present study is to evaluate and classify these herbal medicines according to their safety in pregnancy based on current literature and determine the proportion of women using herbals in each of the safety categories. A second objective was to investigate risk factors associated with the use of potentially hazardous herbal medicines during pregnancy.

## Methods

### Study data and population

This was a multinational, cross-sectional study conducted in 18 countries (Australia, Austria, Canada, Croatia, Finland, France, Iceland, Italy, Norway, Poland, Russia, Serbia, Slovenia, Sweden, Switzerland, The Netherlands, United Kingdom, and USA) between October 1st 2011 and February 29th 2012. Women were eligible to participate if they were pregnant or had at least one child less than 1 year of age. Women were recruited to complete an online-self-reported questionnaire via the placement of banners (invitation to participate in the study) on national websites and/or social networks frequently visited by pregnant women and new mothers. The banners contained information about the rationale behind the study and the criteria for partcipation such as: “Moms-to-be and new moms, share your thoughts in an international survey about your views on medication use in pregnancy. Click here to read more about the study and take part.” In each participating country, the online questionnaire was available for 2 months. The survey questionnaire was administered by Questback (http://www.questback.com). A detailed description of the study design and methods has been published previously [13].

Consent was obtained from each participant. When a woman clicked on the survey link, a description of the study was presented and she was asked whether she was willing to participate. Informed consent was given by ticking a Yes response. The Regional Ethic Committee, Region South-East in Norway, evaluated the study protocol in its multinational aspect and approved the study. Further approvals were provided from the Faculty of Medicine and Health Science Research Ethics Committee of the University of East Anglia in the UK, The National Bioethics Committee in Iceland and The Scientific Ethic Board, and Provincial Health Service of Trento in Italy. Permission to analyze the herbal medicine study data was also obtained from the Research Ethic Board of the Hospital for Sick Children, Toronto, Canada. All data were handled and stored anonymously.

The online questionnaire captured data on maternal health, socio-demographic, and lifestyle characteristics as well as use of herbal and conventional medicines in pregnancy. Maternal characteristics included age, marital status, educational level, mother tongue, employment status, parity, pregnancy intention, and information on use of assisted reproductive technology. Life-style characteristics included folic acid use and smoking status before and during pregnancy and alcohol consumption after awareness of pregnancy.

The following question about use of herbal medicine was posed to all study participants *“Did you take any herbal preparations during pregnancy (e.g. ginger, echinacea, valerian, cranberries)? If yes, please provide the name of all herbal preparations you have taken during pregnancy”*. Herbal medicine use could also be reported under the specific questions about diseases and pregnancy-related health ailments throughout the questionnaire, as described in detail elsewhere [13]. The names of all herbal medicines used during pregnancy were captured as free text. Herbal medicine were defined according to the World Health Organization’s definition: “any medicinal product based on herbs, herbal materials, herbal preparations and finished herbal products, that contain as active ingredients parts of plants, other plant materials, or combinations thereof ” [17]. Medicinal products based on animal components, vitamins, minerals or homeopathic products were not considered herbal medicines.

A pre-determined herbal medicine classification list (common name and Latin name) was compiled by the study team and followed the format of the World Health Organization’s Anatomical Therapeutic Chemical (ATC) code convention as a means to standardize the coding in the questionnaire database. The free text responses were coded according to this pre-determined classification list by the national coordinator in each country. When a product name representing a multi-herbal combination or combination product was entered, an internet search on the product name was performed and the botanical ingredient(s) coded according to the pre-determined classification list. Any mineral supplement(s) and vitamins were recorded separately whenever present in the combination product and excluded from the estimation of herbal use. The form of the herbal medicine (tea, tablet, or tincture) was not specifically requested nor was the dose.

In the survey, women were asked to identify the source(s) of the recommendation to use the herbal medicine. The reported sources were then categorized into the following groups: informal (including the sources: own initiative, family/friends, internet, magazines/media and herbal shop personnel), healthcare provider (including the sources: physician, midwife/nurse and pharmacy personnel), and other (neither informal nor HCP). Women who had responses from both informal and HCP recommendation sources were classified into the “Both informal and HCP group”.

### Determination of the safety classification of herbal medicines

Herbal medicines were categorized into one of four safety categories; safe, caution, contraindicated or unknown (Table 1) based on a summary of the literature, reference textbooks and monographs. The safety classifications were defined as shown in Table 1.

**Table 1:** Overview of herbal medicines used in pregnancy according to safety classification and number of users

Classification	Description	Number of herbs (%)	Number of users (%)
	Total:	126 (100.0)	4,911 (100.00)
Safe	Evidence for the safety of the herb in pregnancy.	28 (22.2)	2,347 (47.8)
Caution	Caution regarding this herb in pregnancy because there is either no or limited human evidence or results suggest that this herb should be used under the supervision of a qualified health care practitioner.	60 (47.6)	1,902 (38.7)
Contraindicated	The use of the herb in pregnancy has demonstrated negative impacts on the pregnancy or fetus.	27 (21.4)	609 (12.4)
Unknown	No reference regarding the use of this herb in pregnancy was found.	11 (8.7)	55 (1.1)

It is important to note that being classified in the “caution” category, does not necessarily imply that the herbal medicine should not be used in pregnancy. Rather it is an indication that either the herbal medicine should be for a specific time period in the pregnancy or under the supervision of a qualified healthcare practitioner

The safety classification was determined by recording the current classification of each herbal medicine from the available reference sources. Several reference sources were reviewed in order to capture different perspectives when classifying the individual herbal medicines: The Australian text book “The Essential Guide to Herbal Safety” [18] The European Medicines Agency’s [19] and North America (Herbal Medicines in Pregnancy & Lactation [15], Botanical Safety Handbook [20], and Botanical Medicine for Women’s Health [21], and Natural Medicines database [22]). If a particular herbal medicine was not listed in the above mentioned reference sources, additional sources were used; Handbook of Medicinal Herbs [23] and PDR for Herbal Medicines [24]. The Handbook of Medicinal Herbs identifies contraindications from multiple sources, often an earlier edition of the Botanical Safety Handbook.

For those herbal medicines which were not listed in any of the referenced books mentioned above, searches in Pubmed, and EBSCO (Alt HealthWatch, AMED, Biomedical Reference Collection, Psychology and Behavioral Sciences Collection, CINHAL Plus, MEDLINE) were performed using first the combination of the terms: “herbal common name and pregnancy” then the “Latin name and pregnancy”. In addition, we performed a search of NCBI’s PubMed database for each of the 125 individual herbal medications reported in the study to identify whether there was evidence of safety in pregnancy. Evidence from controlled studies in human pregnancy was considered first. If no evidence in human studies was available, then *in vivo* research was considered in the classification. The searches were performed from inception to July 2015.

Once the safety classification of each herbal medicine was compiled from the different sources, one author (DAK) reviewed the information and assigned the preliminary classification. The safety classification for each herbal medicine was reviewed by the co-authors (DAK, HN, and Gro Cecilie Havnen) and all discrepancies discussed until agreement was obtained.

We made two assumptions to the safety classifications; 1) that the classification is for the herbal medicine itself rather than a concentrated whole herbal extract form or concentrated isolated constituent of the plant, 2) the dose is assumed to be at therapeutic levels, suitable for oral administration, versus excessive consumption of the herbal medicine or an alternate route of administration. If an herbal medicinal product included several herbs, each herbal was assessed individually and classified.

### Statistical analysis

Factors associated with the use of contraindicated herbal medicines in pregnancy (dichotomous variable: contraindicated herbal medicine user versus non-contraindicated herbal medicine user) were explored via the Generalized Estimating Equations (GEE) [25]. The GEE method is often used to analyze correlated response data such as multiple observations over time for the same individual or clustered data where observations are grouped based on sharing some common characteristic. The use of the GEE in the current study permitted the results to account for any clustering on region of residency. Data are presented as adjusted odds ratios (aOR) with 95 % confidence intervals (CI). A two-tailed *p*-value of <0.05 was considered statistically significant. The multivariate GEE model was developed by first selecting candidate variables whose results, in a univariate model, had a *p*-value < 0.15. Variables with a *p*-value >0.05, no effect, or those having less than a 20 % impact on the beta coefficients of the retained variables were not included in the multivariate model. The final multivariate model included statistically significant independent variables (i.e. employment status, education level, folic acid use during pregnancy, alcohol use during pregnancy, and recommendation source) and potential confounders (i.e. age as a continuous variable). All statistical analyses were performed by using the Statistical Package for the Social Sciences (SPSS) version 20.0 (IBM® SPSS® Statistics).

## Results

Of the 9615 women who indicated their willingness to participate in the study, 9483 (98.6 %) accepted and completed the questionnaire. After exclusion of isolated responses from ineligible countries (*n* = 4), as well as from the Central (*n* = 20) and South (*n* = 346) American regions, we reached a final study population of 9113 women from five different regions and 18 countries. The sample mostly included women from Western (*n* = 3201), Northern (*n* = 2820) or Eastern Europe (*n* = 2342), followed by North America (*n* = 533) and Australia (*n* = 217). A detailed participant flow chart was previously published [13]. The sample was representative of the birthing populations in each participating country with respect to age, parity and smoking habits [13]. However, our sample comprised a greater number of women with high educational levels versus the general birthing population in each country. In total, 29.3 % of the women (*n* = 2673) reported the use of herbal medicines in pregnancy. We were able to identify the specific herbal medicines used by 2379 women (89.0 %). Specific herbals could not be identified for the remaining 294 women (11.0 %), as these women either provided a general response, such as “herbal teas,” the manufacturer’s name rather than that of the herbal medicine or replied that she could not remember. These cases were therefore not included in the safety evaluation.

There were 126 different herbal medicines used in pregnancy, and which could be evaluated according to their safety in pregnancy. Twenty-seven out of 126 herbal medicines were classified as contraindicated in pregnancy and these are detailed in Additional file 1: Table S1a. Of the analzed population of 2379 women, contraindicated herbal medicines were used by 476 women (20.0 %) (Table 1). The most frequently used contraindicated herbal medicines were *Vaccínium vítis-idaéa* (cowberry) (29.8 %), *Levisticum officinale* (lovage) (19.7 %), and *Leonurus cardiaca* (motherwort) (16.6 %). Twenty-eight herbal medicines were classified as safe for use in pregnancy (Additional file 1: Table S1b). These herbal medicines were used by the largest number of women (*n* = 1128, 47.4 %) (Table 1). The most frequently used herbal medicines classified as safe were *Zingiber officinale (*ginger) (56.7 %), *Vaccinium oxycoccus/macrocarpon* (cranberry) (55.0 %)*, and Mentha* x *piperita* (peppermint) (15.9 %)

The greatest number of herbal medicines were classified as requiring caution in pregnancy (Additional file 1: Table S1d). Sixty herbal medicines were used by 751 women (31.6 %) (Table 1). Thirty-six (36/60, 60 %) of the herbal medicines in this group had limited evidence of the safety for use in pregnancy whereas the remaining 24 (40 %) had some evidence of potentially harmful effects in pregnancy. The most frequently used herbal medicines classified as requiring caution in pregnancy were *Valeriana officinalis* (valerian) (*n* = 388), *Rubus idaeus* (raspberry) *(n = 301) and Rosa canina (*dog-rose) (*n* = 148).

There were 11 herbal medicines for which no information on the safety in pregnancy could be found. These herbal medicines were used by only 24 women, representing a small portion of herbal medicine use (1.1 %) (Table 1). These herbs were *Algae* (algae), *Aronia melanocarpa* (black chokeberry), *Bidens tripartita* (water agrimony), *Calluna vulgaris* (heather), *Citricidal* sp*.* (grapefruit extract), *Cucurbita pepo* (cucurbit/squash), *Fagopyrum esculentum* (buckwheat), *Hippophae rhamnoides* (sea buckthorn), *Olea europaea* (olive), *Potentilla reptans* (potentilla), and *Rhodiola rosea* (rhodiola).

**Fig. 1:**
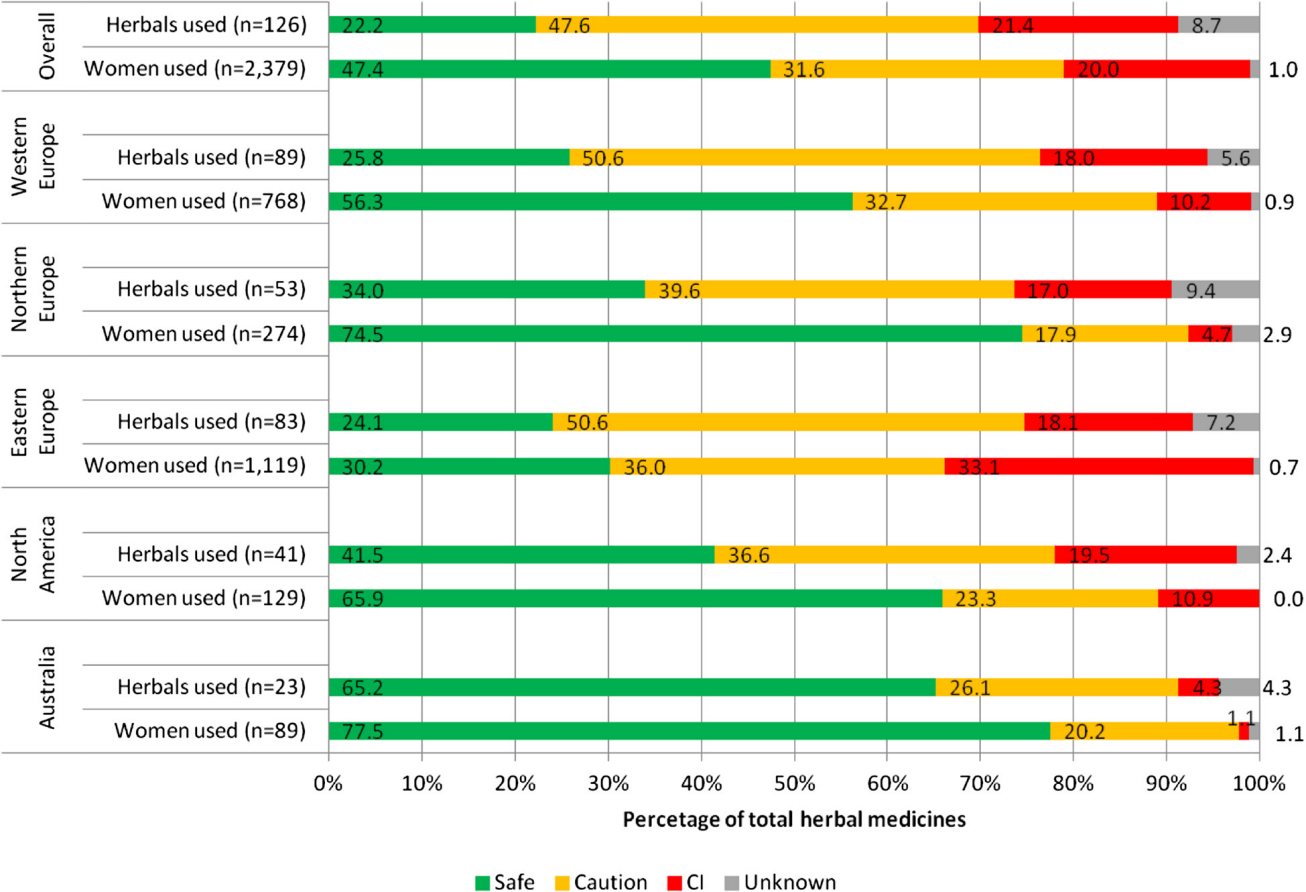
Percentage of herbal medicines used and the number of women who used these herbal medicines by safety classification, overall and by region

There was an important difference between regions in terms of safety of herbal medicines used (Fig. 1). North America had the highest number of herbal medicines that were classified as contraindicated in pregnancy (19.5 %); however, a small number of women (10.9 %) used these herbal medicines. Among the contraindicated herbs used by women in North America were *Cannabis* spp*.* (marijuana) and *Carica papaya* (papaya).

Eastern Europe had the highest number of users of both contraindicated herbal medicines (33.1 %) and “use with caution in pregnancy” category (36 %). Among the contraindicated herbs used by women in Eastern Europe were *Vaccínium vítis-idaéa* (cowberry*)*, *Levisticum officinale* (lovage) and *Leonurus cardiaca* (motherwort). Women in this region exclusively used these herbal medicines.

### Risk factors

Several important maternal factors were found to be associated with the use of contraindicated herbal medicines in pregnancy; namely, working in the home, having a university education, not using folic acid and consuming alcohol. Despite our initial hypothesis that women would inadvertently use contraindicated herbal medicines based on either their own initiative or upon the recommendation from family or friends, we found that the recommendation to take a contraindicated herbal medicine was three times more likely to be from a HCP than an informal source. Factors associated with the use of contraindicated herbal medicines in pregnancy are summarized in Table 2.

**Table 2:** Factors associated with the use of contraindicated herbal medicines in pregnancy

	No use of CI herbals		Use of CI herbals		Total	Adjusted Odds Ratio (95 % CI)
	N	%	N	%		
Age (continuous)						0.95 (0.90–1.01)
Employment status						
Student	183	77.2	54	22.8	237	1.05 (0.87–1.25)
Housewife	163	79.9	41	20.1	204	**1.45 (1.18–1.79)**
HCP	209	80.7	50	19.3	259	1.07 (0.55–2.08)
Job seeker	68	82.9	14	17.1	82	1.00 (0.39–2.58)
None	81	79.4	21	20.6	102	1.02 (0.67–1.54)
Employed other sector	1197	80.2	296	19.8	1493	Reference
Education level						
Primary	66	85.7	11	14.3	77	0.90 (0.43–1.85)
High School	441	83.8	85	16.2	526	Reference
University	1166	77.8	332	22.2	1498	**1.97 (1.60–2.44)**
Other	230	82.7	48	17.3	278	1.18 (1.00–1.39)
Folic acid use before and/or during pregnancy						
Yes	1819	80.1	453	19.9	2272	Reference
No	84	78.5	23	21.5	107	**1.44 (1.14–1.82)**
Alcohol use after awareness of pregnancy						
Yes	393	76.3	122	23.7	515	**1.48 (1.20–1.80)**
Can’t remember	20	71.4	8	28.6	28	1.48 (0.82–2.66)
No	1490	81.2	345	18.8	1835	Reference
Recommendation source						
Informal (family, internet, etc)	780	89.2	94	10.8	874	Reference
Other	24	88.8	3	11.2	27	0.97 (0.29–3.26)
Health care personnel	362	73.7	129	26.3	491	**3.02 (2.65–3.57)**
Both Informal and health care personnel	435	76.2	136	24.8	571	**2.56 (2.35–2.78)**

"Bold data" highlights statistically significant data

## Discussion

To our knowledge, this is the first study to explore the safety of herbal medicines use in pregnancy across several regions of the world. Several of the findings are of clinical importance. Firstly, it is reassuring that the majority of women reported using herbal medicines that were considered safe for use during pregnancy. On the other hand, it is of concern that there still are a substantial number of women using potentially harmful herbal medicines during pregnancy. This is especially worrisome since health care professionals more frequently recommended use of these herbal medicines than other sources. Most of the contraindications were because of evidence of being emmenagogues and/or uterine stimulants (*Caulophyllum thalictroides* (blue cohosh), *Cimicifuga racemosa* (black cohosh), *Capsella bursa*-*pastoris* (shepherd’s purse)) which could result in negative pregnancy outcomes (Table 1). Secondly, it is clear that there are regional differences in herbal traditions. For example, a substantial proportion of women from Eastern Europe exclusively used three herbal medicines. While, the largest group of contraindicated herbal medicine users were those from North America. Clinicians and health care personnel in care of pregnant women in countries with high use of contraindicated herbal medicines in pregnancy should evaluate these findings. Clearly, this represents an opportunity for further detailed investigations with respect to both maternal and neonatal outcomes. Thirdly, this study highlights the urgent need for more data on safety and efficacy of herbal medicine during pregnancy. A recent systematic review on the use of herbal medicine in pregnancy could only identify 14 RCTs evaluating five different herbal medicines [26]. The most studied herbal medicine is *Zingiber officinale* (ginger) with 10 individual studies. The remaining other herbal medicines were *Vaccinium oxycoccus/macrocarpon* (cranberry)*, Hypericum perforatum* (St. John’s wort)*, Rubus idaeus* (raspberry) and *Allium sativum* (garlic) with one study each. This lack of depth in evidence represents a challenge to health care personnel caring for pregnant women.

In several instances, where there was no information on the impact of the herbal medicine in human pregnancy, animal studies were used to determine the classification (see references in Additional file 1: Table S1a-d). However, results from animal studies may not be directly extrapolated to humans [20, 27, 28].

### Challenges classifying herbal safety in pregnancy

This study also presents a practical illustration of how challenging it is to classify safety of herbal medicine. The sources we used gave, at times, different classification recommendations representing different regional traditions or points of view. For example, of the herbal medicines that were included in this study and evaluated by the European Medicines Agency, only two herbal medicines have the status of “may be considered for use” in pregnancy *Psyllii semen/Plantago ovata* (psyllium) [29] and *Salix alba* (white willow) [30]. This legislative approach is not reflected in the classifications presented here. Further, traditionally, herbal medicines are often used in combination. However, existing literature sources do not classify combinations of herbal medicines. Although we classified women using several herbals according to the most harmful herbal used, we did not considered potential synergistic effects or interaction between the herbals. With the increasing prevalence of the use of herbal medicines and their availability as non-prescription products designed for self-selection there is a need for high quality information on the safety in pregnancy of both single and combination herbal medicines.

### Discussion on specific herbals

The herbal medicine, *Rubus idaeus* (raspberry) is classified as “use with caution” in pregnancy despite its long history of traditional and widespread use by women in pregnancy [31]. This classification was done, in part, to highlight the high need for additional research on this herbal medicine and its use in pregnancy. The evidence for its use is currently limited to one RCT and an observational study [26, 31]. Finding from these studies suggest that the intake of raspberry did not have negative fetal or pregnancy outcomes; however, its use did not demonstrate any benefits [31].

With respect to the use of *Vaccinium oxycoccus/macrocarpon* (cranberry), its’ use was classified as “safe for use” in pregnancy. Cranberry is another frequently used herbal medicine for both urinary tract infection (UTI) prophylaxis and treatment [13, 26]. There is evidence from studies with non-pregnant patients that suggests that Cranberry can be helpful for reducing UTI recurrence [26, 32, 33]; however, the evidence to support its effectiveness in UTI treatment in pregnancy is weak [34]. No negative fetal or pregnancy outcomes were identified in a large retrospective cohort study involving 68522 women, of whom 919 used cranberry in pregnancy [35]. However, as UTIs may have negative effects on pregnancy outcomes [36], it is essential that antibiotics are used to treat UTIs and that herbal medicines are not used as alternative to conventional prescribed medication for UTIs in pregnancy.

The herbal medicines *Leonarus cardiaca* (motherwort) and *Levisticum officinale* (lovage) are two herbal medicines that were classified as contraindicated for use in pregnancy. The rationale for this classification is based on the emmenagogue action of these herbal medicines [21, 37]. An emmenagogue is an herbal medicine that stimulates menstrual flow and activity [38]. Sources refer also to motherwort as a nervine (having a potentially beneficial effect on the nervous system [38]) and as a cardiotonic (having observable beneficial action on the heart and blood vessels [38]), while lovage also acts as a bitter (having an impact on the digestive system [38]) which can help to address indigestion and anti-spasmodic actions [38]. Both of these herbal medicines were used by women in Russia only and used by 11 % of herbal users in Russia. The scope of the study did not include the collection of fetal and pregnancy outcomes, dosage, nor frequency of use data, so whether the classification of both of these herbal medicines as contraindicated in pregnancy is appropriate requires further study.

### Strengths and Limitations

This was the first study that uniformly collected information regarding the use of herbal medicines in pregnancy and attempted to classify the herbal medicines used, bringing together perspectives from several sources and countries into the classification. The use of a web-based recruitment strategy enabled us to reach a wide segment of the birthing population. Further, we sought to determine the maternal risk factors for the use of contraindicated herbal medicines, highlighting the need for additional education and research for healthcare practitioners with respect to herbal safety in pregnancy.

There are some limitations to bear in mind regarding this study. Firstly, women were invited to participate via banners posted on pregnancy-related websites. The study design implied no probability sampling of the target population; respondents were those women who happened to have internet access, visited the website(s) where the invitation was posted, and decided to participate in the survey. Hence, the possibility of a self-selection bias cannot be ruled out. On the other hand, the anonymous web-based approach may be especially appropriate for childbearing-age women residing in countries with high internet penetration rates, as it was in this study (range 60 to 97 %) [39]. Since women have been shown to use the internet in a very high extent during pregnancy to search for pregnancy-related information [40, 41] this population is probably a suitable target group in e-epidemiology.

Recent epidemiological studies indicate the validity of web-based recruitment methods [42, 43]. We previously assessed the representativeness of the study participants to the general birthing populations in each study and found that the women in the study had higher education and were slightly more often primiparous than the general birthing populations in the various countries [44]). Moreover, since an online questionnaire was used, it is not possible to calculate a conventional response rate. However, of the women who indicated their willingness to participate, 98.6 % completed the questionnaire.

As in all studies based on self-reported data, the accuracy of our data depends on the accuracy of maternal reporting. Our estimates are likely to be an underestimation of the true prevalence of herbal medicine use in pregnancy as we were dependent upon women to recall which herbal medicines were taken and herbal names were not specifically queried in the questionnaire. We did not capture the plant part, type of extract, dose, dose form or, duration of the use of the herbal medicine in pregnancy. Capturing this data would have permitted more extensive evaluations of contraindication or caution for use. Our results should be interpreted with these strengths and limitations in mind.

## Conclusion

Based on the current literature the majority of women in this study used an herbal medicine that was classified as safe for use in pregnancy. Women who reported taking a contraindicated herb were more likely to have been recommended this use by an HCP rather than informal source(s), indicating an urgent need for more education among HCPs. The paucity of human studies on herbal medicines safety in pregnancy stands in stark contrast to the widespread use of these products among pregnant women.

## Additional file

Additional file 1: Detailed listing of herbal medicines used according to their safety classification. (PDF 1342 kb)

## Abbreviations

aOR: adjusted odds ratio
ATC: anatomical therapeutic chemical
CI: confidence interval
GEE: generalized estimating equation
HCP: healthcare practitioner
RCT: randomized controlled trial
UTI: urinary tract infection
