# Supplementary material for: Safety classification of herbal medicines used in pregnancy in a multinational study
Source: BMC Complement Altern Med. 2016 Mar 15;16:102. doi: 10.1186/s12906-016-1079-z (PMC4793610; doi:10.1186/s12906-016-1079-z)
Supplement: Additional file 1: — Detailed listing of herbal medicines used according to their safety classification. (PDF 1342 kb) [file 12906_2016_1079_MOESM1_ESM.pdf]

Table 1a: Overview of Contraindicated herbal medicines used by the study participants during pregnancy.

Common name	*Latin name*	Number of women using herb in pregnancy	Relevant actions	Comments	Reference Sources accessed
Yarrow	*Achillea millefolium*	13	anti-microbial[1], anti-hemorrhagic[1], urinary antiseptic[1], astringent[1], vulnerary[1], spasmolytic[1]	Concern regarding the thujone content and resulting abortifacient properties.[1] In one reproductive toxicology study in rats, yarrow was associated with reduced fetal weight and increased placental weight; however, yarrow was not materno-toxic.[2]	[1, 3-8]
Garden Angelica	*Angelica archangelica*	1	astringent[9], diuretic[9], vulnerary[9], anti-inflammatory[9]	Traditional history of use as an emmenagogue.[10]	[5, 10, 11]
Dong Quai	*Angelica polymorpha /Angelica sinensis*	2	reproductive tonic[1], anti-thrombotic[1], anti-inflammatory[1]	Of particular concern is in first trimester use and in women with a tendency for spontaneous abortions since there is a substantial risk of causing harm to the fetus.[4] There is also an association with increased bleeding.[1]	[1, 3-5, 10, 12]
Marijuana	*Cannabis* spp	3	sedative[1], spasmolytic[1], anodyne[1], appetite stimulant[1]	There is evidence in some study for both major malformation and increase in preterm birth with the use of marijuana in pregnancy.[13-17] In addition, evidence is emerging regarding the negative impact on the cognitive abilities of children whose mothers' used marijuana in pregnancy.[18-21]	[19, 20, 22-27]
Shepherd's purse	*Capsella bursa-pastoris*	1	anti-hemorrhagic[4], urinary antiseptic[4]	Evidence of both uterine stimulant and emmenagogue activity.[1, 10] Evidence of fetal malformation in *in vivo* studies.[4]	[1, 4, 5, 10, 28, 29]
Papaya	*Carica papaya*	8	anti-spasmodic[30], anti-microbial[30]	Evidence for increased fetal loss and malformations in *in vivo* studies associated with papain.[31, 32]	[10, 33]
Blue Cohosh	*Caulophyllum thalictroides*	2	spasmolytic[4], uterine and ovarian tonic[4], emmenagogue, oxytocic[4]	Several case reports of negative fetal outcomes from the use of blue cohosh in the last weeks of pregnancy.[10, 34-36]	[1, 3-5, 10, 37, 38]
Black Cohosh	*Cimicifuga racemosa*	3	spasmolytic[4], estrogen modulating[4], uterine tonic[4]	Traditionally only used to assist with birth as a component of a partus parapator.[4, 10]	[1, 3-5, 10, 34, 39-41]
Cinnamon	*Cinnamomum aromaticum*	21	astringent[1], carminative[1]	As flavouring, oil is contraindicated, *in vivo* evidence of fetal malformation.[10]	[1, 10, 42, 43]
Licorice	*Glycyrrhiza glabra*	23	adaptogen[1], anti-inflammatory[1], anti-tussive[1], anti-viral[1]	Heavy consumption, >500 mg/week associated with pre-term birth.[44, 45] Studies in the children of mothers' who consumed high doses (>500 mg/wk) of licorice in pregnancy showed altered cortisol levels and cognitive abilities [46, 47] A prospective	[1, 3, 10, 49]

Common name	*Latin name*	Number of women using herb in pregnancy	Relevant actions	Comments	Reference Sources accessed
				study on the impact of licorice in pregnancy found that licorice was not teratogenic [48].	
English Ivy	*Hedera helix*	12	expectorant[30], antispasmodic[30]	Evidence of harmful effects in animal studies, not studied in human pregnancy.[50]	[5, 50, 51]
Motherwort	*Leonurus cardiaca*	79	nervine[1], spasmolytic[1], uterine tonic[1], hypotensive[1], cardiotonic[1], emmenagogue[4]	Evidence of both uterine stimulant and emmenagogue activity.[1, 10] Evidence of fetal malformations in *in vivo* studies.[4]	[1, 4, 5, 10, 52, 53]
Lovage	*Levisticum officinale*	94	anti-inflammatory[30], bitter[30], anti-spasmodic[30]	Uterine stimulant and emmenagogue	[5, 30, 54, 55]
White/Common Horehound	*Marrubium vulgare*	7	expectorant[4], spasmolytic[4], bitter[4]	Traditional use as an emmenagogue.[10] Evidence of fetal malformations in *in vivo* studies.[4]	[4, 10, 56, 57]
Tea-Tree	*Melaleuca alternifolia*	3	anti-microbial[1]		[58]
Butterbur	*Petasites hybridus*	3	anti-inflammatory[30], anti-migraine[30], tonic[30]	Concern regarding the hepatoxicity and mutagenic potential of the pyrrolizidine alkaloids.[1]	[1, 59]
Parsley	*Petroselinum crispum*	55	antioxidant[3], abortifacient[3]	As food, otherwise contraindicated, evidence for abortifacient activity at higher doses.[3]	[3, 10, 60]
Boldo	*Peumus boldus*	2	cholagogue[4], sedative[4], diuretic[4]	Animal studies suggest that boldo is fetotoxic.[4]	[4, 5, 61, 62]
Kava	*Piper methysticum*	1	anxiolytic[4], hypnotic[4]	Concern regarding the hepatoxicity and mutagenic potential of the pyrrolizidine alkaloids.[10]	[1, 3, 4, 10, 63]
White willow	*Salix alba*	8	anti-inflammatory, analgesic	Salicylates do cross the placenta.[64]	[1, 4, 10, 64]
Sage	*Salvia officinalis*	47	anhidrotic[1], estrogenic[1], emmenagogue[1], astringent[1], antiseptic[1], carminative[1], spasmolytic[1]	As food, otherwise contraindicated due to thujone content and the potential abortifacient activity.	[1, 4, 5, 10, 65, 66]
Thuja	*Thuja occidentalis*	2	anti-microbial[4], depurative[4], anti-viral[4], anti-fungal[4]	Associated with increased risk of malformations in humans [4]	[1, 4, 5, 10, 67]
Thyme	*Thymus vulgaris*	30	antimicrobial[4], expectorant[4], spasmolytic[4]	As food, otherwise contraindicated due to concern regarding abortifacient activity.[10]	[4, 10] [1, 5, 68-70]
Fenugreek	*Trigonella foenum-graecum*	1	lactogogue[1], aphrodisiac[1], lipid lowering[1], hypoglycemic[4]	Evidence for anti-fertility and abortifacient activity.[10] Possibility for hypolipidemic and hypothyroid effects.[1]	[1, 3-5, 10, 71]
Coltsfoot	*Tussilago farfara*	6	anti-inflammatory[30], anti-microbial[30], demulcent[30]	Concern regarding the heptoxicity and mutagenic potential of the pyrrolizidine alkaloids.	[10, 72]c
Cowberry	*Vaccínium vítis-idaéa*	142	urinary antiseptic[30], astringent[30]		[30, 73, 74]
Common vervain	*Verbena officinalis*	40	nervine[1], spasmolytic[1], uterine stimulant[1]	Potential for abortifacient and oxytocic effects.[1, 10]	[1, 10, 75]

Table 1b. Overview of safe herbals used by the study participants during pregnancy

Common name	*Latin name*	Number of women using the herb in pregnancy	Relevant actions	Comments	Reference Sources accessed
Horse Chestnut	*Aesculus hippocastanum*	1	venotonic[1], anti-inflammatory[1]	Limited evidence; one study in pregnancy related to leg edema found no adverse effects.[3, 76]	[1, 3, 4, 10, 77-79]
Garlic	*Allium sativum*	78	anti-microbial[1], antihypertensive[1], anti-atherosclerotic[1], lipid lowering[1]	Garlic administered during the third trimester to pregnant women at high risk of pre-eclampsia, was found to reduce hypertension in one RCT study[80], a second study in women at high risk for pre-eclampsia found no impact on pregnancy outcomes [81], and in a cohort study, the consumption of garlic was associated with a 50% reduction in preterm birth [82] .	[1, 3-5, 10, 80, 83]
Aloe vera	*Aloe vera*	23	vulnerary[4], anti-viral[4], anti-inflammatory[4]	Gel: topically use only; Latex/resin CI	[3-5, 10, 84, 85]
Oats	*Avena sativa*	3	nervine[1]		[1, 10, 86, 87]
Bryophyllums	*Bryophyllums*	6	analgesic[30], anti-inflammatory[30], vulnerary[30]	Short term use; studies found for insomnia[88] and as an oxytocic in labour [89].	[88-94]
Green tea	*Camellia sinensis*	12	antiviral[1], antioxidant[1], anti-dyslipidemic[1]	as tea in limited amounts, otherwise contraindicated in high dose due to caffeine content.[1]	[1, 3-5, 10, 95, 96]g
Senna alexandrina	*Cassia acutifolia*	37	laxative[1]		[1, 3-5, 10, 97-99]
German chamomile	*Chamomilla recutita*	177	nervine[1], anti-inflammatory[1], spasmolytic[1], carminative[1], sedative (mild)[1]	As tea in limited amounts. Consistent use has been associated with premature constriction of fetal ductus arteriosus.[100]	[1, 4, 5, 10, 101]
Lemon fruit	*Citrus* sp.	89		As food	
Tumeric	*Curcumae longa*	1	Anti-inflammatory[1]		[1, 3, 4, 10]
Echinacea	*Echinacea* sp.	90	immunomodulator[1], anti-inflammatory[1], anti-microbial[1], alterative[1], lymphatic[1]	No association with an increase in malformation found in one prospective study [102].	[1, 3, 4, 10, 102-108]
Siberian ginseng	*Eleutherococcus senticosus*	3	adaptogen[1], immunomodulator[1]		[1, 3, 4, 10, 109]
Eucalyptus	*Eucalyptus* sp.	31	anti-inflammatory[30], expectorant[30]	external use only	[10, 110-112]
St. John's wort	*Hypericum perforatum*	25	antidepressant[1], antiviral[1], nervine[1], vulnerary[1]	One prospective study no differences in major malformations, and preterm birth with the use of St. John's wort in pregnancy [113].	[3-5, 10, 113-115]
Peppermint	*Mentha* x *piperita*	180	antiemetic[1], spasmolytic[4], carminative[4]	As tea	[1, 3, 4, 10,

Common name	*Latin name*	Number of women using the herb in pregnancy	Relevant actions	Comments	Reference Sources accessed
					116-118]
Ginseng	*Panax ginseng*	2	adaptogen[1], immune modulating[4]		[1, 3-5, 10, 119-121]
Passiflora	*Passiflora incarnata*	21	anxiolytic[4], spasmolytic[4]		[1, 3-5, 10, 122, 123]
Anise	*Pimpinella anisum*	17	spasmolytic[1], carminative[1], lactogogue[1]	As water extracted tea; avoid alcohol extract and oil due to potential anti-implantation activity.[10]	[1, 5, 10, 124-126]
Prunes	*Prunus domestica*	50	laxative	As food	
Psyllium	*Psyllii semen / Plantago ovata*	124	bulk laxative[1]	Possible side effects of gas, bloating with insufficient water[1].	[1, 10, 127-130]
Milk thistle	*Silybum marianum*	1	hepatoprotective[1], antioxidant[1]		[1, 3-5, 10, 131, 132]
Slippery elm	*Ulmus fulva*	2	demulcent[1]		[10] [1, 133]
Nettle	*Urtica dioica / urens*	37	nutritive[1], vascular tonic[1], antiallergic[1], diuretic[1]		[1, 3-5, 10, 134-136]
Bilberry, Huckleberry	*Vaccinium myrtillus*	12	vasoprotective[1], urinary complaints[1]		[4, 10, 137]
Cranberry	*Vaccinium oxycoccus/ macrocarpon*	621	urinary antiseptic[1], astringent[4]	One cohort study investigated cranberry consumption in pregnancy on malformations and outcomes finding no negative impacts [138].	[1, 3, 4, 10, 138-140]
Chaste tree	*Vitex agnus-castus*	1	prolactin inhibitor[4], dopaminergic agonist[4]	For first trimester use only	[1, 3, 4, 10, 141-143]
Ginger	*Zingiber officinale*	640	anti-nauseate[1], antiemetic[1], anti-inflammatory[1], spasmolytic[1], carminative[1], anti-platelet[1]	< 1 g/day[144] One cohort study investigated ginger consumption in pregnancy on malformations and outcomes finding no negative impact.[145] A meta-analysis of ginger in pregnancy found no adverse events associated with its used for nausea and vomiting in pregnancy.[146]	[1, 3-5, 10, 144-150]
Fiber crops	(unspecified)	63	bulk laxative[1]	Possible side effects of gas, bloating with insufficient water[1].	

Table 1c. Overview of herbals with unknown safety in pregnancy used by the study participants during pregnancy.

Common name	*Latin name*	Number of women using herb in pregnancy	Relevant actions	Comment
Algae	*Algae*	5	Nutritive, possible source of vegan omega-3 essential fatty acids[151]	It is difficult to determine specifically what women meant by Algae, since this is a diverse group of organisms, which can include spirulina, chlorella, blue-green algae.
Black chokeberry	*Aronia melanocarpa*	9	Anti-inflammatory[152], hepatoprotective[152]	One study examined the effects of chokeberry on oxidative stress in IUGR[153].The focus was on oxidative stress rather than pregnancy outcomes.
Water agrimony	*Bidens tripartita*	13	diaphoretic[154], astringent[154]	
Heather	*Calluna vulgaris*	1	anti-inflammatory[30], vulnerary[30]	
Grapefruit extract	*Citricidal* sp.	2	Antibacterial[155], antifungal[155], preservative[155]	
Cucurbit/squash	*Cucurbita pepo*	3	anti-inflammatory[30], demulcent[30]	
Buckwheat	*Fagopyrum esculentum*	3	emollient[30], antiedemic[30]	Good source of the flavinoid rutin, which requires caution in pregnancy.[156] [157] However, evidence for buckwheat specifically in pregnancy could not be found.
Sea buckthorn	*Hippophae rhamnoides L.*	11	antioxidant[30], astringent[30], vulnerary[30]	
Olive	*Olea europaea*	2	antiviral[1], antimicrobial[1]	
Potentilla	*Potentilla reptans /Potentilla anserina*	1	antipyretic[30], astringent[30], tonic[30], antiseptic[30], anti-spasmodic[30]	
Rhodiola	*Rhodiola rosea*	5	adaptogen[1], cardiotonic/cardioprotective[1]	

Table 1d. Overview of herbals used by the study participants during pregnancy where caution in pregnancy is recommended

Common name	*Latin name*	Number of women using herb in pregnancy	Relevant actions	Comment	Reference sources accessed
Lady's mantle	*Alchemilla vulgaris*	9	anti-inflammatory[30], diuretic [30], bitter[30]	No toxic constituents identified[10] however, not studied in human pregnancy.	[1, 10, 158]
Marshmallow	*Althea officinalis*	31	demulcent[4], urinary demulcent[4]	No evidence of harmful effects; however, the data is limited.	[1, 4, 10, 159]
Pineapple	*Ananas comosus*	3	anti-inflammatory[30], tonic[30], vulnerary[30]	Conflicting evidence regarding abortifacient effects in mice and rats.[160, 161]	
Uva ursi/bearberry	*Arctostaphylos uva-ursi*	65	urinary antiseptic[1], astringent[1], anti-inflammatory[1]	No evidence of harmful effects; however, the data is limited.	[1, 4, 5, 10, 162, 163]
Wild indigo	*Baptisia tinctoria*	2	anti-inflammatory[30], immunostimulant[30], emmenagogue[30]	Use under the supervision of a qualified heath care practitioner.[10]	[5, 10, 164]
Downy birch, Birch	*Betula pubescens*	49	anti-inflammatory[1], diuretic[1]	No toxic constituents identified[10] however, not studied in human pregnancy.	[1, 5, 10, 165, 166]
Calendula	*Calendula officinalis*	18	vulnerary[1], anti-inflammatory[1], antimicrobial[1], lymphatic[1]	No evidence of harmful effects; however, the data is limited. In one animal study (Wistar rats), negative effects on maternal weight gain were found when an hydroalcoholic mixture was administered in the later stage of pregnancy [167].	[1, 3-5, 10, 168, 169]
Capsicum	*Capsicum* sp.	4	analgesic[30], anti-inflammatory[30], astringent[30]	As food; otherwise caution, in vivo studies suggest anti-implantation activity at higher doses.[10]	[10, 170]
Caraway	*Carum carvi*	6	carminative[1] lactogogue[1]	As food otherwise caution, no toxic constituents identified[10] however, not studied in human pregnancy.	[1, 10, 171]
Seaside Centaury	*Centaurium littorale*	94	hepatic[1]	No toxic constituents identified[10] however, not studied in human pregnancy.	[10, 172, 173]
Iceland moss	*Cetraria islandica*	9	anti-inflammatory, antispasmodic[30], bitter[30]	No toxic constituents identified[10] however, not studied in human pregnancy.	[10, 174]
Rutin	*Constituent/bioflavinoid*	53	Venous insufficiency	There is conflicting evidence, some authors suggest that Rutin can contribute to a miscarriage.	[157, 175-180]
Hawthorne	*Crataegus monogyna*	22	cardiotonic[4], antioxidant[4]	No evidence of harmful effects; however, the data is limited. In one reproductive toxicology study hawthorne demonstrated no adverse effects on rat fetal development at 56 times the human dose [181]	[4, 10, 182]
Artichoke	*Cynara scolymus*	38	lipid lowering[4], hepatoprotective[4], bitter[4], antiemetic[4]	No evidence of harmful effects; however, the data is limited.	[1, 4, 183]

Common name	*Latin name*	Number of women using herb in pregnancy	Relevant actions	Comment	Reference sources accessed
Damiana	*Damianae folium*	1	aphrodisiac[1], nervine tonic[4]	No evidence of harmful effects; however, the data is limited.	[1, 3, 4, 184]
Cardamon	*Elettaria cardamomum*	1	carminative[30], anti-microbial[30], antifungal[30]	As food, otherwise caution, no toxic constituents identified[10] however, not studied in human pregnancy.	[10, 185]
Horsetail	*Equisetum arvense*	9	diuretic [1], urinary astringent[1], anti-inflammatory[1]	No evidence of harmful effects; however, the data is limited.	[1, 4, 10, 186, 187]
Fennel	*Foeniculum vulgare*	58	lactagogue[1], spasmolytic[1], estrogenic[1], carminative[1]	As food, otherwise caution, use under guidance of a qualified healthcare practitioner. Oil and alcohol extracts are not recommended.[10]	[1, 3-5, 10, 188-191]
Gentian	*Gentiana lutea / purpurea*	39	hepatic[4], bitter[4]	No evidence of harmful effects; however, the data is limited.	[1, 3, 4, 10, 192, 193]
Hibiscus	*Hibiscus sabdariffa*	1	antimicrobial[30], demulcent[30]	No evidence of harmful effects animal studies; however, not studied in human pregnancy. An animal study (rats) demonstrated delayed puberty onset in offspring whose mothers' consumed the herb in pregnancy [194].	[10, 195]
Barley	*Hordeum vulgare*	4	Demulcent[30], digestive[30], diuretic[30]	No toxic constituents identified[10] however, not studied in human pregnancy.	[5, 10, 196]
Common hop	*Humulus lupulus*	13	sedative (mild)[1], anxiolytic[1], spasmolytic[1], estrogenic[1]	No evidence of harmful effects; however, the data is limited.	[1, 4, 10, 197, 198]
Walnut	*Juglans nigra / regia*	5	antihelminitic[4], depurative[4], antimicrobial[30]	Juglans nigra: No toxic constituents identified[10] however, not studied in human pregnancy.<br>Juglans regia: unknown	[199]
Bay	*Laurus nobilis*	2	antimicrobial[30], carminative[30]	As food; otherwise caution, not studied in human pregnancy.[10]	[10, 200]
Common Flax	*Linum usitatissimum*	22	phytoestrogen[1], laxative[1], lipid loweri[201]ng[1]	Associated in one study with increase preterm delivery[202], an RCT explored this association finding no difference [203]. Animal studies suggest altered hormonal effects in off-spring, dosing in the 500-1000 mg/kg/day.[1, 204]	[3, 5, 10, 205]
High mallow	*Malva sylvestris*	5	anti-inflammatory[30], antiseptic[30], demulcent[30]	No toxic constituents identified[10] however, not studied in human pregnancy.	[5, 10]
Alfalfa	*Medicago sativa*	5	nutritive[1], phytoestrogen[1]	There are constituents that are believed to have estrogenic[206, 207] [201]and uterine stimulant[208] properties. No human pregnancy	[1, 3, 5, 10, 209]

Common name	*Latin name*	Number of women using herb in pregnancy	Relevant actions	Comment	Reference sources accessed
				data available.	
Lemon Balm	*Melissa officinalis*	83	nerivne[1], spasmolytic[1], anxiolytic[1], anti-viral[1]	No toxic constituents identified[10] however, not studied in human pregnancy.	[1, 3, 4, 10, 210, 211]
Squaw vine	*Mitchela repens*	1	uterine astringent/tonic[1]	No evidence of harmful effects; however, the data is limited. Traditionally used to prevent threatened abortions and before delivery as a partus preparater.[10, 212, 213]	[3, 5, 10]
Nasturtium (watercress)	*Nasturtium officinalis*	1	antimicrobial[30], anti-inflammatory[30], vulnerary[30]	As food; otherwise caution limited evidence. [10]	[10, 214]
Catnip	*Nepeta cataria*	2	spasmolytic[1], nervine[1], sedative (mild)[1]	No evidence of harmful effects animal studies; however, not studied in human pregnancy.	[1, 5, 10, 215]
Basil	*Ocimum basilicum*	5	antibacterial[30], antioxidant[30], anti-inflammatory[30]	As food; otherwise caution, not studied in human pregnancy.[10]	[5, 10, 216]b
Evening primrose	*Oenothera biennis*	16	anti-inflammatory[1], high in gamma-linolenic acid[1]	The data is limited; however, one study reported an increased risk of pregnancy complications.[3, 217]	[1, 4, 10, 218]
Marjoram	*Origanum majorana*	1	anti inflammatory[30], antispasmodic[30], carminative[30]	As food otherwise caution, no toxic constituents identified[10] however, not studied in human pregnancy.	[10, 219]
Oregano	*Origanum vulgare*	1	analgesic[30], anti-inflammatory[30]	As food, otherwise caution due to limited evidence.[10]	[1, 5, 10, 220]
Kidney tea/Java tea	*Orthosiphon stamineus*	17	antispasmodic[30], anti-inflammatory[30]	One study found on reproductive toxicology in rats suggested that the herbal medicine may contain androgenic compounds.[221] Another study suggested that it may have an impact on male and female fertility.[222]	
Black pepper	*Piper nigrum*	2	antiseptic[30], carminative[30], tonic[30]	As food; otherwise caution, in vivo studies suggest anti-implantation activity at higher doses.[10]	[5, 10, 223]
Plantain	*Plantago major*	7	antimicrobial[30], vulnerary[30]	No toxic constituents identified[10] however, not studied in human pregnancy.	[10, 224]
Primula	*Primula* sp.	43	antioxidant[30], anti-inflammatory[30], antispasmodic[30]	No toxic constituents identified[10] however, not studied in human pregnancy.	[10, 225-227]
Oak	*Quercus robur*	4	astringent[1]	No toxic constituents identified[10] however, not studied in human pregnancy. *Quercus alba* is safe for topical use[1]	[10, 228, 229]
Black currant	*Ribes nigrum*	48	analgesic[30], anti-inflammatory[30], diuretic[30]	No toxic constituents identified[10] however, not studied in human pregnancy.	[10, 230, 231]

Common name	*Latin name*	Number of women using herb in pregnancy	Relevant actions	Comment	Reference sources accessed
Dog-rose	*Rosa majalis /Rosa canina*	148	antioxidant[30], antispasmodic[30]	No toxic constituents identified[10] however, not studied in human pregnancy.	[10, 232]
Rosemary	*Rosmarinus officinalis*	98	spasmolytic[1], circulatory stimulant[1], antioxidant[1], anti-microbial[1], anti-inflammatory[1]	As food, otherwise caution due to limited evidence.[4, 10]	[1, 4, 5, 10, 233-235]
Raspberry	*Rubus idaeus folicum*	301	uterine tonic[1], astringent[1], partus preparator[1], nutritive[1]	No evidence of harmful effects; however, the data is limited. One RCT demonstrated no difference in expected labor parameters.[236]	[1, 3-5, 10, 237-239]
Curled Dock	*Rumex crispus*	36	aperients[1], alerative[1]	No evidence of harmful effects; however, the data is limited.	[1, 4, 10]
Butcher's broom	*Ruscus aculeatus*	1	anti-inflammatory[4], venotonic[4]	No evidence of harmful effects; however, the data is limited.	[4, 10, 240, 241]
Elder, Black elder	*Sambucus nigra, Sambucus* sp.	48	diaphoretic[4], anticatarrhal[4]	No evidence of harmful effects; however, the data is limited.	[4, 10, 242, 243]
Skullcap	*Scutellaria lateriflora*	2	nervine, spasmolytic, mild sedative	No evidence of harmful effects; however, the data is limited.	[1, 4, 5, 10, 244]
European goldenrot/woundwort	*Solidago virgaurea*	5	diuretic[1], anti-inflammatory[1]	No evidence of harmful effects; however, the data is limited.	[1, 4, 5, 10, 245]
Cloves	*Syzygium aromaticum*	15	antimicrobial[30], anti-inflammatory[30], antispasmodic[30]	As food; otherwise caution, limited evidence[10]	[10, 246, 247]
Dandelion	*Taraxacum officinale*	8	diuretic[1], bitter tonic[1], cholerectic[1]	As food; otherwise caution, limited evidence.[4]	[1, 4, 10, 248-250]
Lime	*Tila europa*	31	sedative (mild) [1], spasmolytic[1], diuretic[1]	No evidence of harmful effects; however, the data is limited.	[1, 4, 10, 251]
Red clover	*Trifolium pratense*	3	phystoestrogen[1], alterative[1], spasmolytic[1]	No toxic constituents identified[10] however, not studied in human pregnancy. Standardized isoflavone products should not be used in pregnancy due to estrogenic activity[1]	[1, 3, 5, 10, 252]
Squill	*Urginea scilla*	2	diuretic[30], hypotensive[30]	One study, using a variant of Urginea, demonstrated neural toxicity in chick embryos.[253]	[254]
Valerian	*Valeriana officinalis*	388	sedative[1], anxiolytic[1], spasmolytic[1], carminative[1]	No evidence of harmful effects; however, the data is limited. A reproductive screening study of valerian in rats demonstrated no maternal toxicity or adverse effects on fetal development [255].	[3, 4, 10, 256, 257]
Mullein	*Verbascum thapsus*	1	expectorant[4], anticatarrhal[4]	No evidence of harmful effects; however, the data is limited.	[1, 4, 10, 258, 259]

Common name	*Latin name*	Number of women using herb in pregnancy	Relevant actions	Comment	Reference sources accessed
Viburnum	*Viburnum opulus/ prunifolium*	2	spasmolytic[4], mild sedative**[4]**, hypotensive**[4]**	No evidence of harmful effects; however, the data is limited. Long history of traditional use for threatened miscarriage and as a partus preprator.[1, 4]	[1, 4, 10]
Violet	*Viola odorata*	8	anti-inflammatory[30], nervine[30]	No toxic constituents identified[10] however, not studied in human pregnancy.	[10, 260]
Common Grape Vine	*Vitis vinifera*	3	antioxidant[30], astringent[30]	No evidence of harmful effects animal studies; however, not studied in human pregnancy.[261]	[261]
Corn silk	*Zea mays*	2	diuretic[4], urinary demulcent[4]	No evidence of harmful effects; however, the data is limited.	[1, 4, 10, 262]

Note: It is important to note that being classified in the "caution" category, does not necessarily imply that the herbal medicine should not be used in pregnancy. Rather it is an indication that either the herbal medicine should be for a specific time period in the pregnancy or under the supervision of a qualified healthcare practitioner.

References

1. Romm AJ: **Botanical medicine for women's health**. St. Louis, Mo.: Churchill Livingstone/Elsevier; 2010: xix, 694 p.

2. Boswell-Ruys CL, Ritchie HE, Brown-Woodman PD: **Preliminary screening study of reproductive outcomes after exposure to yarrow in the pregnant rat**. *Birth Defects Res B Dev Reprod Toxicol* 2003, **68**(5):416-420.

3. Mills E, Duguoa J-J, Perri D, Koren G: **Herbal medicines in pregnancy and lactation: An evidence-based approach**. London ; New York: Taylor & Francis; 2006.

4. Mills SY, Bone K: **The essential guide to herbal safety**. St. Louis, Mo.: Elsevier Churchill Livingstone; 2005.

5. Brinker FJ: **Herb contraindications and drug interactions**, 4th edn. Sandy, Or.: Eclectic Medical Publications; 2010.

6. Committee on Herbal Medicinal Products: **Community herbal monograph on achillea millefolium l., herba** London: European Medicines Agency; 2011

7. Committee on Herbal Medicinal Products: **Community herbal monograph on achillea millefolium l., flos**. London: European Medicines Agency; 2011

8. Yarrow. In: Natural Medicine. 2015. https://naturalmedicines.therapeuticresearch.com/databases/food,-herbs-supplements/professional.aspx?productid=151. Accessed 17 Jul 2015

9. Hoffmann D: **Medical herbalism : The science and practice of herbal medicine**. Rochester, Vt.: Healing Arts Press; 2003.

10. Gardner ZE, McGuffin M, American Herbal Products Association.: **American herbal products association's botanical safety handbook**, 2nd edn. Boca Raton: American Herbal Products Association, CRC Press; 2013.

11. Angelica. In: Natural Medicine. 2015. https://naturalmedicines.therapeuticresearch.com/databases/food,-herbs-supplements/professional.aspx?productid=281. Accessed 17 Jul 2015

12. Dong quai. In: Natural Medicine. 2015. https://naturalmedicines.therapeuticresearch.com/databases/food,-herbs-supplements/professional.aspx?productid=936. Accessed 17 Jul 2015

13. Linn S, Schoenraum SC, Monson RR, Rosner R, Stubblefield PC,Ryan KJ: **The association of marijuana use with outcome of pregnancy.** *American Journal of Public Health* 1983, **73**(10):1161-1164.

14. Saurel-Cubizolles MJ, Prunet C, Blondel B: **Cannabis use during pregnancy in france in 2010**. *BJOG: An International Journal of Obstetrics & Gynaecology* 2014, **121**(8):971-977.

15. Burns L, Mattick RP, Cooke M: **The use of record linkage to examine illicit drug use in pregnancy**. *Addiction* 2006, **101**(6):873-882.

16. van Gelder MM, Reefhuis J, Caton AR, Werler MM, Druschel CM, Roeleveld N: **Characteristics of pregnant illicit drug users and associations between cannabis use and perinatal outcome in a population-based study**. *Drug & Alcohol Dependence* 2010, **109**(1-3):243-247.

17. Fried PA: **The consequences of marijuana use during pregnancy: A review of the human literature**. *Journal of Cannabis Therapeutics* 2002, **2**(3/4):85-104.

18. Sundram S: **Cannabis and neurodevelopment: Implications for psychiatric disorders**. *Human Psychopharmacology: Clinical & Experimental* 2006, **21**(4):245-254.

19. Wu CS, Jew CP, Lu HC: **Lasting impacts of prenatal cannabis exposure and the role of endogenous cannabinoids in the developing brain**. *Future Neurol* 2011, **6**(4):459-480.

20. Jaques SC, Kingsbury A, Henshcke P, Chomchai C, Clews S, Falconer J, Abdel-Latif ME, Feller JM, Oei JL: **Cannabis, the pregnant woman and her child: Weeding out the myths**. *J Perinatol* 2014, **34**(6):417-424.

21. Marroun HE, Hudziak JJ, Tiemeier H, Creemers H, Steegers EA, Jaddoe VW, Hofman A, Verhulst FC, van den Brink W, Huizink AC: **Intrauterine cannabis exposure leads to more aggressive behavior and attention problems in 18-month-old girls**. *Drug & Alcohol Dependence* 2011, **118**(2-3):470-474.

22. Desai A, Mark K, Terplan M: **Marijuana use and pregnancy: Prevalence, associated behaviors, and birth outcomes**. *Obstet Gynecol* 2014, **123 Suppl 1**:46S.

23. Johnson EM: **Substance abuse and women's health**. *Public Health Rep* 1987, **102**(4 Suppl):42-48.

24. Reece AS: **Chronic toxicology of cannabis**. *Clin Toxicol (Phila)* 2009, **47**(6):517-524.

25. Schempf AH: **Illicit drug use and neonatal outcomes: A critical review**. *Obstet Gynecol Surv* 2007, **62**(11):749-757.

26. Williams JH, Ross L: **Consequences of prenatal toxin exposure for mental health in children and adolescents: A systematic review**. *Eur Child Adolesc Psychiatry* 2007, **16**(4):243-253.

27. Marijuana. In: Natural Medicine. 2015. https://naturalmedicines.therapeuticresearch.com/databases/food,-herbs-supplements/professional.aspx?productid=947. Accessed 17 Jul 2015

28. Committee on Herbal Medicinal Products: **Community herbal monograph on capsella bursa-pastoris (l.) medikus, herba** London: European Medicines Agency; 2011

29. Shepherd's purse. In: Natural Medicine. 2015. https://naturalmedicines.therapeuticresearch.com/databases/food,-herbs-supplements/professional.aspx?productid=51. Accessed 17 Jul 2015

30. Duke JA, Duke JA: **Handbook of medicinal herbs**, 2nd edn. Boca Raton, FL: CRC Press; 2002.

31. Oderinde O, Noronha C, Oremosu A, Kusemiju T, Okanlawon OA: **Abortifacient properties of aqueous extract of carica papaya (linn) seeds on female sprague-dawley rats**. *Niger Postgrad Med J* 2002, **9**(2):95-98.

32. Facts and Comparisons (Firm): **The review of natural products**. St. Louis, Mo.: Facts and Comparisons; 1999: v.

33. Papaya. In: Natural Medicine. 2015. https://naturalmedicines.therapeuticresearch.com/databases/food,-herbs-supplements/professional.aspx?productid=488. Accessed 17 Jul 2015

34. Gunn TR, Wright IM: **The use of black and blue cohosh in labour**. *N Z Med J* 1996, **109**(1032):410-411.

35. Jones TK, Lawson BM: **Profound neonatal congestive heart failure caused by maternal consumption of blue cohosh herbal medication**. *J Pediatr* 1998, **132**(3 Pt 1):550-552.

36. Finkel RS, Zarlengo KM: **Blue cohosh and perinatal stroke**. *N Engl J Med* 2004, **351**(3):302-303.

37. Dugoua JJ, Perri D, Seely D, Mills E, Koren G: **Safety and efficacy of blue cohosh (caulophyllum thalictroides) during pregnancy and lactation**. *Can J Clin Pharmacol* 2008, **15**(1):e66-73.

38. Blue cohosh. In: Natural Medicine. 2015. https://naturalmedicines.therapeuticresearch.com/databases/food,-herbs-supplements/professional.aspx?productid=987. Accessed 17 Jul 2015

39. Committee on Herbal Medicinal Products: **Community herbal monograph on cimicifuga racemosa (l.) nutt., rhizoma** London: European Medicines Agency; 2010

40. Dugoua JJ, Seely D, Perri D, Koren G, Mills E: **Safety and efficacy of black cohosh (cimicifuga racemosa) during pregnancy and lactation**. *Can J Clin Pharmacol* 2006, **13**(3):e257-261.

41. Black cohosh. In: 2015. https://naturalmedicines.therapeuticresearch.com/databases/food,-herbs-supplements/professional.aspx?productid=857. Accessed 17 Jul 2015

42. Committee on Herbal Medicinal Products: **Community herbal monograph on cinnamomum verum j.S. Presl, cortex, rhizoma** London: European Medicines Agency; 2011

43. Cinnamon. In: Natural Medicine. 2015. https://naturalmedicines.therapeuticresearch.com/databases/food,-herbs-supplements/professional.aspx?productid=330. Accessed 17 Jul 2015

44. Strandberg TE, Andersson S, Jarvenpaa AL, McKeigue PM: **Preterm birth and licorice consumption during pregnancy**. *Am J Epidemiol* 2002, **156**(9):803-805.

45. Strandberg TE, Jarvenpaa AL, Vanhanen H, McKeigue PM: **Birth outcome in relation to licorice consumption during pregnancy**. *Am J Epidemiol* 2001, **153**(11):1085-1088.

46. Raikkonen K, Pesonen AK, Heinonen K, Lahti J, Komsi N, Eriksson JG, Seckl JR, Jarvenpaa AL, Strandberg TE: **Maternal licorice consumption and detrimental cognitive and psychiatric outcomes in children**. *Am J Epidemiol* 2009, **170**(9):1137-1146.

47. Raikkonen K, Seckl JR, Heinonen K, Pyhala R, Feldt K, Jones A, Pesonen AK, Phillips DI, Lahti J, Jarvenpaa AL, Eriksson JG, Matthews KA, Strandberg TE, Kajantie E: **Maternal prenatal licorice consumption alters hypothalamic-pituitary-adrenocortical axis function in children**. *Psychoneuroendocrinology* 2010, **35**(10):1587-1593.

48. Choi JS, Han JY, Ahn HK, Ryu HM, Kim MY, Chung JH, Nava-Ocampo AA, Koren G: **Fetal and neonatal outcomes in women reporting ingestion of licorice (glycyrrhiza uralensis) during pregnancy**. *Planta Med* 2013, **79**(2):97-101.

49. Licorice. In: Natural Medicine. 2015. https://naturalmedicines.therapeuticresearch.com/databases/food,-herbs-supplements/professional.aspx?productid=881. Accessed 17 Jul 2015

50. Committee on Herbal Medicinal Products: **Community herbal monograph on hedera helix l. , folium** London: European Medicines Agency; 2011

51. English ivy. In: Natural Medicine. 2015. https://naturalmedicines.therapeuticresearch.com/databases/food,-herbs-supplements/professional.aspx?productid=465. Accessed 17 Jul 2015

52. Committee on Herbal Medicinal Products: **Community herbal monograph on leonurus cardiaca l., herba** London: European Medicines Agency; 2010

53. Motherwort. In: Natural Medicine. 2015. https://naturalmedicines.therapeuticresearch.com/databases/food,-herbs-supplements/professional.aspx?productid=126. Accessed 17 Jul 2015

54. Committee on Herbal Medicinal Products: **Community herbal monograph on levisticum officinale koch, radix**. London: European Medicines Agency; 2012

55. Lovage. In: Natural Medicine. 2015. https://naturalmedicines.therapeuticresearch.com/databases/food,-herbs-supplements/professional.aspx?productid=725. Accessed 17 Jul 2015

56. Committee on Herbal Medicinal Products: **Community herbal monograph on marrubium vulgare l.,herba**. London: European Medicines Agency; 2013

57. White horehound. In: Natural Medicine. 2015. https://naturalmedicines.therapeuticresearch.com/databases/food,-herbs-supplements/professional.aspx?productid=886. Accessed 17 Jul 2015

58. Tea tree oil. In: Natural Medicine. 2015. https://naturalmedicines.therapeuticresearch.com/databases/food,-herbs-supplements/professional.aspx?productid=113. Accessed 17 Jul 2015

59. Butterbur. In: Natural Medicine. 2015. https://naturalmedicines.therapeuticresearch.com/databases/food,-herbs-supplements/professional.aspx?productid=649. Accessed 17 Jul 2015

60. Parsley. In: Natural Medicine. 2015. https://naturalmedicines.therapeuticresearch.com/databases/food,-herbs-supplements/professional.aspx?productid=792. Accessed 17 Jul 2015

61. Committee on Herbal Medicinal Products: **Community monogrpah on peumus boldus molina, folium**. London: European Medicines Agency; 2009

62. Boldo. In: 2015. https://naturalmedicines.therapeuticresearch.com/databases/food,-herbs-supplements/professional.aspx?productid=593. Accessed 17 Jul 2015

63. Kava. In: Natural Medicine. 2015. https://naturalmedicines.therapeuticresearch.com/databases/food,-herbs-supplements/professional.aspx?productid=872. Accessed 17 Jul 2015

64. Committee on Herbal Medicinal Products: **Community herbal monograph on salix, cortex**. London: European Medicines Agency; 2009

65. Committee on Herbal Medicinal Products: **Community herbal monograph on salvia officinalis l., folium** London: European Medicines Agency; 2009

66. Sage. In: Natural Medicine. 2015. https://naturalmedicines.therapeuticresearch.com/databases/food,-herbs-supplements/professional.aspx?productid=504. Accessed 17 Jul 2015

67. Thuja. In: Natural Medicine. 2015. https://naturalmedicines.therapeuticresearch.com/databases/food,-herbs-supplements/professional.aspx?productid=1117. Accessed 17 Jul 2015

68. Committee on Herbal Medicinal Products: **Community herbal monograph on thymus vulgaris l ., thymus zygis loefl. Ex. L., aetheroleum** London: European Medicines Agency; 2010

69. Committee on Herbal Medicinal Products: **Community herbal monograph on thymus vulgaris l ., thymus zygis l., herba**. London: European Medicines Agency; 2013

70. Thyme. In: Natural Medicine. 2015. https://naturalmedicines.therapeuticresearch.com/databases/food,-herbs-supplements/professional.aspx?productid=823. Accessed 17 Jul 2015

71. Fenugreek. In: Natural Medicine. 2015. https://naturalmedicines.therapeuticresearch.com/databases/food,-herbs-supplements/professional.aspx?productid=733. Accessed 17 Jul 2015

72. Coltsfoot. In: Natural Medicine. 2015. https://naturalmedicines.therapeuticresearch.com/databases/food,-herbs-supplements/professional.aspx?productid=730. Accessed 17 Jul 2015

73. Lingonberry. In: Natural Medicine. 2015. https://naturalmedicines.therapeuticresearch.com/databases/food,-herbs-supplements/professional.aspx?productid=813. Accessed 17 Jul 2015

74. Gruenwald J, Brendler T, Jaenicke C: **Pdr for herbal medicines**, 4th edn. Montvale, NJ: Medical Economic Company; 2000.

75. Verbena. In: Natural Medicine. 2015. https://naturalmedicines.therapeuticresearch.com/databases/food,-herbs-supplements/professional.aspx?productid=88. Accessed 17 Jul 2015

76. Steiner M: **Untersuchungen zur odemvermindernden und odemportektiven wirking von ro kastaniensamenextrakt.** . *Phlebol Prokto* 1990, **19**:239-242.

77. Committee on Herbal Medicinal Products: **Community herbal monograph on aesculus hippocastanum l., semen**. London: European Medicines Agency; 2009

78. Committee on Herbal Medicinal Products: **Community herbal monograph on aesculus hippocastanum l., cortex**. London: European Medicines Agency; 2011

79. Horse chestnut. In: Natural Medicine. 2015. https://naturalmedicines.therapeuticresearch.com/databases/food,-herbs-supplements/professional.aspx?productid=1055. Accessed 17 Jul 2015

80. Ziaei S, Hantoshzadeh S, Rezasoltani P, Lamyian M: **The effect of garlic tablet on plasma lipids and platelet aggregation in nulliparous pregnants at high risk of preeclampsia**. *Eur J Obstet Gynecol Reprod Biol* 2001, **99**(2):201-206.

81. Aalami-Harandi R, Karamali M, Asemi Z: **The favorable effects of garlic intake on metabolic profiles, hs-crp, biomarkers of oxidative stress and pregnancy outcomes in pregnant women at risk for pre-eclampsia: Randomized, double-blind, placebo-controlled trial**. *J Matern Fetal Neonatal Med* 2014:1-8.

82. Myhre R, Brantsaeter AL, Myking S, Eggesbo M, Meltzer HM, Haugen M, Jacobsson B: **Intakes of garlic and dried fruits are associated with lower risk of spontaneous preterm delivery**. *J Nutr* 2013, **143**(7):1100-1108.

83. Garlic. In: Natural Medicine. 2015. https://naturalmedicines.therapeuticresearch.com/databases/food,-herbs-supplements/professional.aspx?productid=300. Accessed 17 Jul 2015

84. Committee on Herbal Medicinal Products: **Community herbal monograph on aloe barbadensis miller and on aloe (various species, mainly aloe ferox miller and its hybrids**. London: European Medicines Agency; 2006

85. Aloe. In: Natural Medicine. 2015. https://naturalmedicines.therapeuticresearch.com/databases/food,-herbs-supplements/professional.aspx?productid=19. Accessed July 17, 2015

86. Committee on Herbal Medicinal Products: **Community herbal monograph on avena sativa l., herba** London: European Medicines Agency; 2008

87. Oats. In: Natural Medicine. 2015. https://naturalmedicines.therapeuticresearch.com/databases/food,-herbs-supplements/professional.aspx?productid=814. Accessed 17 Jul 2015

88. Lambrigger-Steiner C, Simoes-Wust AP, Kuck A, Furer K, Hamburger M, von Mandach U: **Sleep quality in pregnancy during treatment with bryophyllum pinnatum: An observational study**. *Phytomedicine* 2014, **21**(5):753-757.

89. Plangger N, Rist L, Zimmermann R, von Mandach U: **Intravenous tocolysis with bryophyllum pinnatum is better tolerated than beta-agonist application**. *Eur J Obstet Gynecol Reprod Biol* 2006, **124**(2):168-172.

90. Gwehenberger B, Rist L, Huch R, von Mandach U: **Effect of bryophyllum pinnatum versus fenoterol on uterine contractility**. *Eur J Obstet Gynecol Reprod Biol* 2004, **113**(2):164-171.

91. Pal S, Sen T, Chaudhuri AK: **Neuropsychopharmacological profile of the methanolic fraction of bryophyllum pinnatum leaf extract**. *J Pharm Pharmacol* 1999, **51**(3):313-318.

92. Simoes-Wust AP, Graos M, Duarte CB, Brenneisen R, Hamburger M, Mennet M, Ramos MH, Schnelle M, Wachter R, Worel AM, von Mandach U: **Juice of bryophyllum pinnatum (lam.) inhibits oxytocin-induced increase of the intracellular calcium concentration in human myometrial cells**. *Phytomedicine* 2010, **17**(12):980-986.

93. Simoes-Wust AP, Jeschke E, Mennet M, Schnelle M, Matthes H, von Mandach U: **Prescribing pattern of bryophyllum preparations among a network of anthroposophic physicians**. *Forsch Komplementmed* 2012, **19**(6):293-301.

94. Wachter R, Brenneisen R, Hamburger M, Mennet M, Schnelle M, Worel AM, Simoes-Wust AP, von Mandach U: **Leaf press juice from bryophyllum pinnatum (lamarck) oken induces myometrial relaxation**. *Phytomedicine* 2011, **19**(1):74-82.

95. Committee on Herbal Medicinal Products: **Community herbal monograph on camellia sinensis (l.) kuntze, non fermentatum folium**. London: European Medicines Agency; 2013

96. Green tea. In: Natural Medicine. 2015. https://naturalmedicines.therapeuticresearch.com/databases/food,-herbs-supplements/professional.aspx?productid=960. Accessed 17 Jul 2015

97. Committee on Herbal Medicinal Products: **Community herbal monograph on cassia senna l., fructus and cassia angustifolia vahl, fructus** London: European Medicines Agency; 2006

98. Committee on Herbal Medicinal Products: **Community herbal monograph on cassia senna l. And cassia angustifolia vahl, folium**. London: European Medicines Agency; 2006

99. Senna. In: Natural Medicine. 2015. https://naturalmedicines.therapeuticresearch.com/databases/food,-herbs-supplements/professional.aspx?productid=652. Accessed 17 Jul 2015

100. Sridharan S, Archer N, Manning N: **Premature constriction of the fetal ductus arteriosus following the maternal consumption of camomile herbal tea**. *Ultrasound Obstet Gynecol* 2009, **34**(3):358-359.

101. German chamomille. In: Natural Medicine. 2015. https://naturalmedicines.therapeuticresearch.com/databases/food,-herbs-supplements/professional.aspx?productid=951. Accessed 17 Jul 2015

102. Gallo M, Sarkar M, Au W, Pietrzak K, Comas B, Smith M, Jaeger TV, Einarson A, Koren G: **Pregnancy outcome following gestational exposure to echinacea: A prospective controlled study**. *Arch Intern Med* 2000, **160**(20):3141-3143.

103. Committee on Herbal Medicinal Products: **Community herbal monograph on echinacea angustifolia dc., radix**. London: European Medicines Agency; 2008

104. Committee on Herbal Medicinal Products: **Community herbal monograph on echinacea purpurea (l.) moench, herba recens** London: European Medicines Agency; 2008

105. Committee on Herbal Medicinal Products: **Community herbal monograph on echinacea pallida (nutt.) nutt., radix** London: European Medicines Agency; 2009

106. Committee on Herbal Medicinal Products: **Community herbal monograph on echinacea purpurea (l.) moench, radix** London: European Medicines Agency; 2010

107. Perri D, Dugoua JJ, Mills E, Koren G: **Safety and efficacy of echinacea (echinacea angustafolia, e. Purpurea and e. Pallida) during pregnancy and lactation**. *Can J Clin Pharmacol* 2006, **13**(3):e262-267.

108. Echinacea. In: Natural Medicine. 2015. https://naturalmedicines.therapeuticresearch.com/databases/food,-herbs-supplements/professional.aspx?productid=981. Accessed 17 Jul 2015

109. Committee on Herbal Medicinal Products: **Community herbal monograph on eleutherococcus senticosus (rupr. Et maxim.) maxim., radix** London: European Medicines Agency; 2008

110. Committee on Herbal Medicinal Products: **Community herbal monograph on eucalyptus globulus l abill ., folium}** London: European Medicines Agency; 2013

111. Committee on Herbal Medicinal Products: **Cmmunity herbal monograph on eucalyptus globulus labill.,eucalyptus polybractea r.T. Baker and /or eucalyptus smithii r.T. Baker, atheroleum**. London: European Medicines Agency; 2014

112. Eucalyptus. In: Natural Medicine. 2015. https://naturalmedicines.therapeuticresearch.com/databases/food,-herbs-supplements/professional.aspx?productid=700. Accessed 17 Jul 2015

113. Moretti ME, Maxson A, Hanna F, Koren G: **Evaluating the safety of st. John's wort in human pregnancy**. *Reprod Toxicol* 2009, **28**(1):96-99.

114. Committee on Herbal Medicinal Products: **Community herbal monograph on hypericum perforatum l. , herba** London: European Medicines Agency; 2009

115. St. John's wort. In: Natural Medicine. 2015. https://naturalmedicines.therapeuticresearch.com/databases/food,-herbs-supplements/professional.aspx?productid=329. Accessed 17 Jul 2015

116. Committee on Herbal Medicinal Products: **Community herbal monograph on mentha x piperita l., folium** London: European Medicines Agency; 2008

117. Committee on Herbal Medicinal Products: **Community herbal monograph on mentha x piperita l., aetheroleum** London: European Medicines Agency; 2007

118. Peppermint. In: Natural Medicine. 2015. https://naturalmedicines.therapeuticresearch.com/databases/food,-herbs-supplements/professional.aspx?productid=705. Accessed 17 Jul 2015

119. Committee on Herbal Medicinal Products: **Community herbal monograph on panax ginseng c.A.Meyer, radix** London: European Medicines Agency; 2014

120. Seely D, Dugoua JJ, Perri D, Mills E, Koren G: **Safety and efficacy of panax ginseng during pregnancy and lactation**. *Can J Clin Pharmacol* 2008, **15**(1):e87-94.

121. Panax ginseng. In: Natural Medicine. 2015. https://naturalmedicines.therapeuticresearch.com/databases/food,-herbs-supplements/professional.aspx?productid=1000. Accessed 17 Jul 2015

122. Committee on Herbal Medicinal Products: **Community herbal monograph on passiflora incarnata l.,herba**. London: European Medicines Agency; 2014

123. Passionflower. In: Natural Medicine. 2015. https://naturalmedicines.therapeuticresearch.com/databases/food,-herbs-supplements/professional.aspx?productid=871. Accessed 17 Jul 2015

124. Committee on Herbal Medicinal Products: **Community herbal monograph on pimpinella anisum l., aetheroleum**. London: European Medicines Agency; 2013

125. Committee on Herbal Medicinal Products: **Community herbal monograph on pimpinella anisum l., fructus**. London: European Medicines Agency; 2013

126. Anise. In: 2015. https://naturalmedicines.therapeuticresearch.com/databases/food,-herbs-supplements/professional.aspx?productid=582. Accessed July 17, 2015

127. Committee on Herbal Medicinal Products: **Community herbal monograph on plantago afra l. Et plantago indica l. , semen**. London: European Medicines Agency; 2013

128. Committee on Herbal Medicinal Products: **Community herbal monograph on plantago ovata forssk., seminis tegumentum**. London: European Medicines Agency; 2013

129. Committee on Herbal Medicinal Products: **Community herbal monograph on plantago ovata forssk., semen**. London: European Medicines Agency; 2013

130. Blond psyllium. In: Natural Medicine. 2015. https://naturalmedicines.therapeuticresearch.com/databases/food,-herbs-supplements/professional.aspx?productid=866. Accessed 17 Jul 2015

131. Milic N, Milosevic N, Suvajdzic L, Zarkov M, Abenavoli L: **New therapeutic potentials of milk thistle (silybum marianum)**. *Nat Prod Commun* 2013, **8**(12):1801-1810.

132. Milk thistle. In: Natural Medicine. 2015. https://naturalmedicines.therapeuticresearch.com/databases/food,-herbs-supplements/professional.aspx?productid=138. Accessed 17 Jul 2015

133. Slippery elm. In: Natural Medicine. 2015. https://naturalmedicines.therapeuticresearch.com/databases/food,-herbs-supplements/professional.aspx?productid=978. Accessed 17 Jul 2015

134. Committee on Herbal Medicinal Products: **Community herbal monograph on urtica dioica l.; urtica urens l., folium** London: European Medicines Agency; 2010

135. Committee on Herbal Medicinal Products: **Community herbal monograph on urtica dioica l. And urtica urens l., herba** London: European Medicines Agency; 2008

136. Stinging nettle. In: Natural Medicine. 2015. https://naturalmedicines.therapeuticresearch.com/databases/food,-herbs-supplements/professional.aspx?productid=664. Accessed 17 Jul 2015

137. Bilberry. In: 2015. https://naturalmedicines.therapeuticresearch.com/databases/food,-herbs-supplements/professional.aspx?productid=202. Accessed 17 Jul 2015

138. Heitmann K, Nordeng H, Holst L: **Pregnancy outcome after use of cranberry in pregnancy--the norwegian mother and child cohort study**. *BMC Complement Altern Med* 2013, **13**:345.

139. Dugoua JJ, Seely D, Perri D, Mills E, Koren G: **Safety and efficacy of cranberry (vaccinium macrocarpon) during pregnancy and lactation**. *Can J Clin Pharmacol* 2008, **15**(1):e80-86.

140. Cranberry. In: Natural Medicine. 2015. https://naturalmedicines.therapeuticresearch.com/databases/food,-herbs-supplements/professional.aspx?productid=958. Accessed 17 Jul 2015

141. Committee on Herbal Medicinal Products: **Community herbal monograph on vitex agnus-castus l., fructus** London: European Medicines Agency; 2010

142. Dugoua JJ, Seely D, Perri D, Koren G, Mills E: **Safety and efficacy of chastetree (vitex agnus-castus) during pregnancy and lactation**. *Can J Clin Pharmacol* 2008, **15**(1):e74-79.

143. Vitex agnus-castus. In: Natural Medicine. 2015. https://naturalmedicines.therapeuticresearch.com/databases/food,-herbs-supplements/professional.aspx?productid=968. Accessed 17 Jul 2015

144. Ding M, Leach M, Bradley H: **The effectiveness and safety of ginger for pregnancy-induced nausea and vomiting: A systematic review**. *Women Birth* 2013, **26**(1):e26-30.

145. Heitmann K, Nordeng H, Holst L: **Safety of ginger use in pregnancy: Results from a large population-based cohort study**. *Eur J Clin Pharmacol* 2013, **69**(2):269-277.

146. Viljoen E, Visser J, Koen N, Musekiwa A: **A systematic review and meta-analysis of the effect and safety of ginger in the treatment of pregnancy-associated nausea and vomiting**. *Nutr J* 2014, **13**:20.

147. Committee on Herbal Medicinal Products: **Community herbal monograph on zingiber officinale roscoe, rhizoma**. London: European Medicines Agency; 2012

148. Borrelli F, Capasso R, Aviello G, Pittler MH, Izzo AA: **Effectiveness and safety of ginger in the treatment of pregnancy-induced nausea and vomiting**. *Obstet Gynecol* 2005, **105**(4):849-856.

149. Fischer-Rasmussen W, Kjaer SK, Dahl C, Asping U: **Ginger treatment of hyperemesis gravidarum**. *Eur J Obstet Gynecol Reprod Biol* 1991, **38**(1):19-24.

150. Ginger. In: Natural Medicine. 2015. https://naturalmedicines.therapeuticresearch.com/databases/food,-herbs-supplements/professional.aspx?productid=961. Accessed 17 Jul 2015

151. Kapoor R, Mehta U: **Effect of supplementation of blue green alga (spirulina) on outcome of pregnancy in rats**. *Plant Foods for Human Nutrition* 1993, **43**(1):29-35.

152. Kokotkiewicz A, Jaremicz Z, Luczkiewicz M: **Aronia plants: A review of traditional use, biological activities, and perspectives for modern medicine**. *J Med Food* 2010, **13**(2):255-269.

153. Pawlowicz P, Wilczynski J, Stachowiak G, Hincz P: **[administration of natural anthocyanins derived from chokeberry retardation of idiopathic and preeclamptic origin. Influence on metabolism of plasma oxidized lipoproteins: The role of autoantibodies to oxidized low density lipoproteins]**. *Ginekol Pol* 2000, **71**(8):848-853.

154. M W: **The earthwise herbal : A complete guide to old world medicinal plants.** Berkeley, Calif.: : North Atlantic Books; 2008.

155. Mills S, Bone K: **Principles and practice of phytotherapy : Modern herbal medicine**. Edinburgh ; New York: Churchill Livingstone; 2000.

156. Kishore K: **Rutin (natural bioflavonoid): Traditional and medicial uses**. *Pharmacologyonline*. vol. 1; 2010: 931937

157. Dar MA, Tabassum N: **Rutin - a potent natural thrombolytic agent**. *International Current Pharmaceutical Journal* 2012, **1**(12):431-435.

158. Alchemilla. In: Natural Medicine. 2015. https://naturalmedicines.therapeuticresearch.com/databases/food,-herbs-supplements/professional.aspx?productid=654. Accessed 17 Jul 2015

159. Committee on Herbal Medicinal Products: **Community herbal monograph on althaea officinalisl., radix** London: European Medicines Agency; 2009

160. Pakrashi A, Basak B: **Abortifacient effect of steroids from ananas comosus and their analogues on mice**. *Journal of Reproduction and Fertility* 1976, **46**(2):461-462.

161. Yakubu MT, Olawepo OJ, Fasoranti GA: **Ananas comosus: Is the unripe fruit juice an abortifacient in pregnant wistar rats?** *Eur J Contracept Reprod Health Care* 2011, **16**(5):397-402.

162. Committee on Herbal Medicinal Products: **Community herbal monograph on arctostaphylos uva-ursi (l.) spreng., folium**. London: European Medicines Agency; 2012

163. Uva ursi. In: Natural Medicine. 2015. https://naturalmedicines.therapeuticresearch.com/databases/food,-herbs-supplements/professional.aspx?productid=350. Accessed 17 Jul 2015

164. Wild indigo. In: Natural Medicine. 2015. https://naturalmedicines.therapeuticresearch.com/databases/food,-herbs-supplements/professional.aspx?productid=600. Accessed 17 Jul 2015

165. Committee on Herbal Medicinal Products: **Community herbal monograph on betula pendula roth; betula pubescens ehrh., folium** London: European Medicines Agency; 2008

166. Birch. In: Natural Medicine. 2015. https://naturalmedicines.therapeuticresearch.com/databases/food,-herbs-supplements/professional.aspx?productid=352. Accessed 17 Jul 2015

167. Silva EJ, Costa-Silva JH, Evêncio LB, Fraga Mdo C, Coelho MC, Wanderley AG: **Reproductive assessment of hydroalcohol extract of calendula officinalis l. In wistar rats**. *Phytotherapy Research* 2009, **23**(10):1392-1398.

168. Committee on Herbal Medicinal Products: **Community herbal monograph on calendula officinalis l., flos** London: European Medicines Agency; 2008

169. Calendula. In: Natural Medicine. 2015. https://naturalmedicines.therapeuticresearch.com/databases/food,-herbs-supplements/professional.aspx?productid=235. Accessed 17 Jul 2015

170. Capsicum. In: Natural Medicine. 2015. https://naturalmedicines.therapeuticresearch.com/databases/food,-herbs-supplements/professional.aspx?productid=945. Accessed 17 Jul 2015

171. Caraway. In: Natural Medicine. 2015. https://naturalmedicines.therapeuticresearch.com/databases/food,-herbs-supplements/professional.aspx?productid=204. Accessed 17 Jul 2015

172. Committee on Herbal Medicinal Products: **Community herbal monograph on centaurium erythraea rafn, herba** London: European Medicines Agency; 2009

173. Centuary. In: Natural Medicine. 2015. https://naturalmedicines.therapeuticresearch.com/databases/food,-herbs-supplements/professional.aspx?productid=399. Accessed 17 Jul 2015

174. Iceland moss. In: Natural Medicine. 2015. https://naturalmedicines.therapeuticresearch.com/databases/food,-herbs-supplements/professional.aspx?productid=516. Accessed 17 Jul 2015

175. Marhic C: **[clinical and rheological efficacy of troxerutin in obstetric gynecology]**. *Rev Fr Gynecol Obstet* 1991, **86**(2 Pt 2):209-212.

176. Wijayanegara H, Mose JC, Achmad L, Sobarna R, Permadi W: **A clinical trial of hydroxyethylrutosides in the treatment of haemorrhoids of pregnancy**. *J Int Med Res* 1992, **20**(1):54-60.

177. Sohn C, Jahnichen C, Bastert G: **[effectiveness of beta-hydroxyethylrutoside in patients with varicose veins in pregnancy]**. *Zentralbl Gynakol* 1995, **117**(4):190-197.

178. Lefebvre G, Lacombe C: **[venous insufficiency in the pregnant woman. Rheological correction by troxerutin]**. *Rev Fr Gynecol Obstet* 1991, **86**(2 Pt 2):206-208.

179. Bamigboye AA, Smyth R: **Interventions for varicose veins and leg oedema in pregnancy**. *The Cochrane Database Of Systematic Reviews* 2007(1):CD001066.

180. Rutin. In: Natural Medicine. 2015. https://naturalmedicines.therapeuticresearch.com/databases/food,-herbs-supplements/professional.aspx?productid=270. Accessed 17 Jul 2015

181. Yao M, Ritchie HE, Brown-Woodman PD: **A reproductive screening test of hawthorn**. *Journal of Ethnopharmacology* 2008, **118**(1):127-132.

182. Hawthorn. In: Natural Medicine. 2015. https://naturalmedicines.therapeuticresearch.com/databases/food,-herbs-supplements/professional.aspx?productid=527. Accessed 17 Jul 2015

183. Artichoke. In: 2015. https://naturalmedicines.therapeuticresearch.com/databases/food,-herbs-supplements/professional.aspx?productid=842. Accessed July 17, 2015

184. Damiana. In: Natural Medicine. 2015. https://naturalmedicines.therapeuticresearch.com/databases/food,-herbs-supplements/professional.aspx?productid=703. Accessed 17 Jul 2015

185. Cardamon. In: 2015. https://naturalmedicines.therapeuticresearch.com/databases/food,-herbs-supplements/professional.aspx?productid=614. Accessed 17 Jul 2015

186. Committee on Herbal Medicinal Products: **Community herbal monograph on equisetum arvense l., herba** London: European Medicines Agency; 2008

187. Horsetail. In: Natural Medicine. 2015. https://naturalmedicines.therapeuticresearch.com/databases/food,-herbs-supplements/professional.aspx?productid=843. Accessed 17 Jul 2015

188. Committee on Herbal Medicinal Products: **Community herbal monograph on foeniculum vulgare miller subsp. Vulgare var. Vulgare , fructus** London: European Medicines Agency; 2007

189. Committee on Herbal Medicinal Products: **Community herbal monograph on foeniculum vulgare miller subsp. Vulgare var. Vulgare , aetheroleum** London: European Medicines Agency; 2007

190. Committee on Herbal Medicinal Products: **Community herbal monograph on foeniculum vulgare miller subsp. Vulgare var. Dulce (miller) thellung, fructus** London: European Medicines Agency; 2007

191. Fennel. In: Natural Medicine. 2015. https://naturalmedicines.therapeuticresearch.com/databases/food,-herbs-supplements/professional.aspx?productid=311. Accessed 17 Jul 2015

192. Committee on Herbal Medicinal Products: **Community herbal monograph on gentiana lutea l., radix** London: European Medicines Agency; 2009

193. Gentian. In: Natural Medicine. 2015. https://naturalmedicines.therapeuticresearch.com/databases/food,-herbs-supplements/professional.aspx?productid=716. Accessed 17 Jul 2015

194. Iyare EE, Adegoke OA: **Mechanism of the delayed puberty onset in offspring of rats that consumed aqueous extract of hibiscus sabdariffa during pregnancy**. *Niger J Physiol Sci* 2008, **23**(1-2):71-77.

195. Hibiscus. In: Natural Medicine. 2015. https://naturalmedicines.therapeuticresearch.com/databases/food,-herbs-supplements/professional.aspx?productid=211. Accessed 17 Jul 2015

196. Barley. In: 2015. https://naturalmedicines.therapeuticresearch.com/databases/food,-herbs-supplements/professional.aspx?productid=799. Accessed July 17, 2015

197. Committee on Herbal Medicinal Products: **Community herbal monograph on humulus lupulus l., flos** London: European Medicines Agency; 2008

198. Hops. In: Natural Medicine. 2015. https://naturalmedicines.therapeuticresearch.com/databases/food,-herbs-supplements/professional.aspx?productid=856. Accessed 17 Jul 2015

199. Committee on Herbal Medicinal Products: **Community herbal monograph on juglans regia l., folium**. London: European Medicines Agency; 2013

200. Bay. In: Natural Medicine. 2015. https://naturalmedicines.therapeuticresearch.com/databases/food,-herbs-supplements/professional.aspx?productid=685. Accessed 17 Jul 2015

201. Newall CA AL, Philpson JD: **Herbal medicine: A guide for healthcare professionals.** London, UK: : The Pharmaceutical Press; 1996.

202. Moussally K, Oraichi D, Berard A: **Herbal products use during pregnancy: Prevalence and predictors**. *Pharmacoepidemiol Drug Saf* 2009, **18**(6):454-461.

203. Knudsen VK, Hansen HS, Osterdal ML, Mikkelsen TB, Mu H, Olsen SF: **Fish oil in various doses or flax oil in pregnancy and timing of spontaneous delivery: A randomised controlled trial**. *BJOG* 2006, **113**(5):536-543.

204. Zanwar AA, Aswar UM, Hegde MV, Bodhankar SL: **Estrogenic and embryo-fetotoxic effects of ethanol extract of linum usitatissimum in rats**. *Journal of Complementary & Integrative Medicine* 2010, **7**(1):17p.

205. Flaxseed. In: Natural Medicine. 2015. https://naturalmedicines.therapeuticresearch.com/databases/food,-herbs-supplements/professional.aspx?productid=991. Accessed 17 Jul 2015

206. Leung AY, Foster S: **Encyclopedia of common natural ingredients used in food, drugs, and cosmetics**, 2nd edn. New York: Wiley; 1996.

207. Elakovich SD, Hampton JM: **Analysis of coumestrol, a phytoestrogen, in alfalfa tablets sold for human consumption**. *J Agric Food Chem* 1984, **32**(1):173-175.

208. Farnsworth NR, Bingel AS, Cordell GA, Crane FA, Fong HH: **Potential value of plants as sources of new antifertility agents i**. *J Pharm Sci* 1975, **64**(4):535-598.

209. Alfalfa. In: 2015. https://naturalmedicines.therapeuticresearch.com/databases/food,-herbs-supplements/professional.aspx?productid=19. Accessed July 17, 2015

210. Committee on Herbal Medicinal Products: **Community herbal monograph on melissa officinalis l., folium**. London: European Medicines Agency; 2013

211. Lemon balm. In: Natural Medicine. 2015. https://naturalmedicines.therapeuticresearch.com/databases/food,-herbs-supplements/professional.aspx?productid=437. Accessed 17 Jul 2015

212. Jody E. Noé, Mary Bove, Janel K: **Herbal tonic formulas for naturopathic obstetrics** *Alternative and Complementary Therapies* 2002, **8**(6):327-335.

213. Ellingwood F: **The american materia medica, therpautics and pharmacognosy**. Evanston, IL: Ellingwoods's Therapeutist.; 1919.

214. Nasturtium. In: Natural Medicine. 2015. https://naturalmedicines.therapeuticresearch.com/databases/food,-herbs-supplements/professional.aspx?productid=526
Accessed 17 Jul 2015

215. Catnip. In: Natural Medicine. 2015. https://naturalmedicines.therapeuticresearch.com/databases/food,-herbs-supplements/professional.aspx?productid=831. Accessed 17 Jul 2015

216. Basil. In: 2015. https://naturalmedicines.therapeuticresearch.com/databases/food,-herbs-supplements/professional.aspx?productid=303. Accessed July 17, 2015

217. Dove D, Johnson P: **Oral evening primrose oil: Its effect on length of pregnancy and selected intrapartum outcomes in low-risk nulliparous women**. *J Nurse Midwifery* 1999, **44**(3):320-324.

218. Committee on Herbal Medicinal Products: **Community herbal monograph on oenothera biennis l.; oenothera lamarckiana l., oleum** London: European Medicines Agency; 2011

219. Marjoram. In: Natural Medicine. 2015. https://naturalmedicines.therapeuticresearch.com/databases/food,-herbs-supplements/professional.aspx?productid=563. Accessed 17 Jul 2015

220. Oregano. In: Natural Medicine. 2015. https://naturalmedicines.therapeuticresearch.com/databases/food,-herbs-supplements/professional.aspx?productid=644. Accessed 17 Jul 2015

221. Muhammad H, Sulaiman SA, Ismail Z, Paumgartten FJR: **Study on the developmental toxicity of a standardized extract of orthosiphon stamineus in rats**. *Revista Brasileira de Farmacognosia* 2013, **23**:513-520.

222. Al-Yahya AA: **Reproductive toxicity of orthosiphon stamineus benth (java tea) in swiss albino mice**. *British Journal of Pharmacology and Toxicology* 2013, **4**(5):181-187.

223. Black pepper. In: 2015. https://naturalmedicines.therapeuticresearch.com/databases/food,-herbs-supplements/professional.aspx?productid=800. Accessed 17 Jul 2015

224. Great plantain. In: Natural Medicine. 2015. https://naturalmedicines.therapeuticresearch.com/databases/food,-herbs-supplements/professional.aspx?productid=677. Accessed 17 Jul 2015

225. Committee on Herbal Medicinal Products: **Community herbal monograph on primula veris l. And/or primula elatior (l.) hill, flos**. London: European Medicines Agency; 2012

226. Committee on Herbal Medicinal Products: **Community herbal monograph on primula veris l. And/or primula elatior (l.) hill, radix**. London: European Medicines Agency; 2012

227. Cowslip. In: Natural Medicine. 2015. https://naturalmedicines.therapeuticresearch.com/databases/food,-herbs-supplements/professional.aspx?productid=65. Accessed 17 Jul 2015

228. Committee on Herbal Medicinal Products: **Community herbal monograph on quercus robur l., quercus petraea .(matt.) liebl., quercus pubescens willd., cortex** London: European Medicines Agency; 2010

229. Oak bark. In: Natural Medicine. 2015. https://naturalmedicines.therapeuticresearch.com/databases/food,-herbs-supplements/professional.aspx?productid=498. Accessed 17 Jul 2015

230. Committee on Herbal Medicinal Products: **Community herbal monograph on ribes nigrum l., folium** London: European Medicines Agency; 2009

231. Ribes nigrum. In: 2015. https://naturalmedicines.therapeuticresearch.com/databases/food,-herbs-supplements/professional.aspx?productid=1085. Accessed 17 Jul 2015

232. Rose hip. In: Natural Medicine. 2015. https://naturalmedicines.therapeuticresearch.com/databases/food,-herbs-supplements/professional.aspx?productid=839. Accessed 17 Jul 2015

233. Committee on Herbal Medicinal Products: **Community herbal monograph on rosmarinus officinalis l., aetheroleum** London: European Medicines Agency; 2010

234. Committee on Herbal Medicinal Products: **Community herbal monograph on rosmarinus officinalis l., folium** London: European Medicines Agency; 2010

235. Rosemary. In: Natural Medicine. 2015. https://naturalmedicines.therapeuticresearch.com/databases/food,-herbs-supplements/professional.aspx?productid=154. Accessed 17 Jul 2015

236. Simpson M, Parsons M, Greenwood J, Wade K: **Raspberry leaf in pregnancy: Its safety and efficacy in labor**. *J Midwifery Womens Health* 2001, **46**(2):51-59.

237. Committee on Herbal Medicinal Products: **Community herbal monograph on rubus idaeus l., folium** London: European Medicines Agency; 2014

238. Holst L, Haavik S, Nordeng H: **Raspberry leaf--should it be recommended to pregnant women?** *Complement Ther Clin Pract* 2009, **15**(4):204-208.

239. Red raspberry. In: Natural Medicine. 2015. https://naturalmedicines.therapeuticresearch.com/databases/food,-herbs-supplements/professional.aspx?productid=309. Accessed 17 Jul 2015

240. Committee on Herbal Medicinal Products: **Community herbal monograph on ruscus aculeatus l., rhizoma** London: European Medicines Agency; 2008

241. Butcher's broom. In: Natural Medicine. 2015. https://naturalmedicines.therapeuticresearch.com/databases/food,-herbs-supplements/professional.aspx?productid=320. Accessed 17 Jul 2015

242. Committee on Herbal Medicinal Products: **Community herbal monograph on sambucus nigra l., flos**. London: European Medicines Agency; 2008

243. Elderberry. In: Natural Medicine. 2015. https://naturalmedicines.therapeuticresearch.com/databases/food,-herbs-supplements/professional.aspx?productid=434. Accessed 17 Jul 2015

244. Skullcap. In: Natural Medicine. 2015. https://naturalmedicines.therapeuticresearch.com/databases/food,-herbs-supplements/professional.aspx?productid=986. Accessed 17 Jul 2015

245. Committee on Herbal Medicinal Products: **Community herbal monograph on solidago virgaurea l., herba** London: European Medicines Agency; 2008

246. Committee on Herbal Medicinal Products: **Community herbal monograph on syzygium aromaticum (l.) merill et l. M. Perry, floris aetheroleum** London: European Medicines Agency; 2011

247. Cloves. In: Natural Medicine. 2015. https://naturalmedicines.therapeuticresearch.com/databases/food,-herbs-supplements/professional.aspx?productid=251. Accessed 17 Jul 2015

248. Committee on Herbal Medicinal Products: **Community herbal monograph on taraxacum officinale weber ex wigg., folium** London: European Medicines Agency; 2009

249. Committee on Herbal Medicinal Products: **Community herbal monograph on taraxacum officinale weber ex wigg., radix cum herba** London: European Medicines Agency; 2009

250. Dandeloin. In: 2015. https://naturalmedicines.therapeuticresearch.com/databases/food,-herbs-supplements/professional.aspx?productid=706. Accessed 17 Jul 2015

251. Committee on Herbal Medicinal Products: **Community herbal monograph on tilia cordata miller, tilia platyphyllos scop. , tilia x vulgaris heyne or their mixtures, flos**. London: European Medicines Agency; 2012

252. Red clover. In: Natural Medicine. 2015. https://naturalmedicines.therapeuticresearch.com/databases/food,-herbs-supplements/professional.aspx?productid=308. Accessed 17 Jul 2015

253. Marx J, Pretorius E, Bester MJ: **Effects of urginea sanguinea, a traditional asthma remedy, on embryo neuronal development**. *Journal of Ethnopharmacology* 2006, **104**(3):315-321.

254. Squill. In: Natural Medicine. 2015. https://naturalmedicines.therapeuticresearch.com/databases/food,-herbs-supplements/professional.aspx?productid=743. Accessed 17 Jul 2015

255. Yao M, Ritchie HE, Brown-Woodman PD: **A developmental toxicity-screening test of valerian**. *J Ethnopharmacol* 2007, **113**(2):204-209.

256. Committee on Herbal Medicinal Products: **Community herbal monograph on valeriana officinalis l., radix**. London: European Medicines Agency; 2006

257. Valerian. In: Natural Medicine. 2015. https://naturalmedicines.therapeuticresearch.com/databases/food,-herbs-supplements/professional.aspx?productid=870. Accessed 17 Jul 2015

258. Committee on Herbal Medicinal Products: **Community herbal monograph on verbascum thapsus l., v. Densiflor umbertol. And v. Phlomoides l., flos**. London: European Medicines Agency; 2008

259. Mullein. In: Natural Medicine. 2015. https://naturalmedicines.therapeuticresearch.com/databases/food,-herbs-supplements/professional.aspx?productid=572. Accessed 17 Jul 2015

260. Committee on Herbal Medicinal Products: **Community herbal monograph on viola tricolor l. And/or subspecies viola arvensis murray (gaud) and viola vulgaris koch (oborny), herba cum flore** London: European Medicines Agency; 2010

261. Committee on Herbal Medicinal Products: **Community herbal monograph on vitis vinifera l., folium**. London: European Medicines Agency; 2010

262. Corn silk. In: Natural Medicine. 2015. https://naturalmedicines.therapeuticresearch.com/databases/food,-herbs-supplements/professional.aspx?productid=140. Accessed 17 Jul 2015
